# IL-17 mediates protective immunity against nasal infection with *Bordetella pertussis* by mobilizing neutrophils, especially Siglec-F^+^ neutrophils

**DOI:** 10.1038/s41385-021-00407-5

**Published:** 2021-05-11

**Authors:** Lisa Borkner, Lucy M. Curham, Mieszko M. Wilk, Barry Moran, Kingston H. G. Mills

**Affiliations:** Immune Regulation Research Group, School of Biochemistry and Immunology, Trinity Biomedical Sciences Institute, Trinity College Dublin, Dublin, Ireland

## Abstract

Understanding the mechanism of protective immunity in the nasal mucosae is central to the design of more effective vaccines that prevent nasal infection and transmission of *Bordetella pertussis*. We found significant infiltration of IL-17-secreting CD4^+^ tissue-resident memory T (T_RM_) cells and Siglec-F^+^ neutrophils into the nasal tissue during primary infection with *B. pertussis*. *Il17A*^−/−^ mice had significantly higher bacterial load in the nasal mucosae, associated with significantly reduced infiltration of Siglec-F^+^ neutrophils. Re-infected convalescent mice rapidly cleared *B. pertussis* from the nasal cavity and this was associated with local expansion of IL-17-producing CD4^+^ T_RM_ cells. Depletion of CD4 T cells from the nasal tissue during primary infection or after re-challenge of convalescent mice significantly delayed clearance of bacteria from the nasal mucosae. Protection was lost in *Il17A*^−/−^ mice and this was associated with significantly less infiltration of Siglec-F^+^ neutrophils and antimicrobial peptide (AMP) production. Finally, depletion of neutrophils reduced the clearance of *B. pertussis* following re-challenge of convalescent mice. Our findings demonstrate that IL-17 plays a critical role in natural and acquired immunity to *B. pertussis* in the nasal mucosae and this effect is mediated by mobilizing neutrophils, especially Siglec-F^+^ neutrophils, which have high neutrophil extracellular trap (NET) activity.

## Introduction

The incidence of pertussis or whooping cough, the respiratory infectious disease caused by the Gram-negative aerobic coccobacillus *Bordetella pertussis* has been on the rise during the last decades, despite high vaccine coverage. The re-emergence of pertussis in certain countries has been linked with a switch from whole-cell pertussis (wP) to acellular pertussis (aP) vaccines in the 1990s.^1,2^ The aP vaccines, which comprise 2–5 *B. pertussis* antigens, including filamentous hemagglutinin (FHA), inactivated pertussis toxin (PT), pertactin (PRN) and fimbriae-2/3, formulated with alum as the adjuvant, have less side effects than wP vaccines,^1^ but appear not to be as effective. Increasing susceptibility in vaccinated children and adolescents has been linked with waning immunity, especially a decline in circulating antibodies after immunization with aP vaccines.^3–5^ Evasion of antibody responses induced by aP vaccines may also have resulted through emergence of *B. pertussis* strains with mutations or deletions in key antigens, especially PT or PRN.^6–10^ However, these aspects alone are not enough to explain the rising incidence of pertussis cases in infants.

Analysis of the periodicity of *B. pertussis* infections showed that the natural cycle was lengthened following the introduction of wP vaccines, but went back to the pre-vaccination length with the introduction of aP vaccines, suggesting that aP vaccines while protecting against severe disease, did not interrupt asymptomatic transmission.^2,11,12^ This is consistent with studies in baboon and mouse models, which have demonstrated that natural infection, and to a lesser extent immunization with wP vaccines, confer protection against nasal infection, whereas immunization with aP vaccines does not. Indeed, there is some suggestion that nasal infection may be enhanced post challenge of baboons or mice immunized with aP vaccines.^13,14^

Immunization of infants with aP vaccines generates strong serum IgG antibody responses against the vaccine antigens and protects against severe pertussis disease for up to 2 years.^15,16^ Studies on antibody responses in infants exposed to *B. pertussis* in the household showed that those with high titers specific for PRN and PT were protected better against whooping cough.^17–19^ However, there is limited evidence of a direct correlation between serum antibodies and protection against *B. pertussis* infection. Studies of T cell responses in peripheral blood of vaccinated children showed that wP vaccines induced antigen-specific IFN-γ production, while aP vaccines predominantly induced the Th2 cytokines IL-4 and IL-5.^20–24^ Peripheral blood CD4 T cells from convalescent children mainly produced IFN-γ, but not IL-5, suggesting that wP immunization like natural *B. pertussis* infection preferentially induces Th1-type responses.^25,26^

Studies in a mouse model have demonstrated that *B. pertussis* infection induced a strong Th1/Th17 response and promoted migration of tissue-resident CD4 T cells (T_RM_) to the respiratory tract, whereas immunization with aP vaccines induced a Th2-polarized response, but no significant respiratory T_RM_ cells.^14^ Early studies on the mechanism of protective immunity demonstrated that IFN-γ plays a crucial role in the clearance of *B. pertussis* from the lungs.^27,28^ We have also reported that IL-17 is required for protection against primary infection of the lungs with *B. pertussis*. IL-17-secreting CD4 T_RM_ cells are induced in lung and nasal tissue following infection.^14,29^ However, a definitive role for IFN-γ or IL-17-secreting T cells in protection against nasal infection has not been established. A full understanding of the mechanism of protection in the nasal cavity is essential for the design of vaccines that prevent nasal as well as lung infection with *B. pertussis*.

In this study, we examined the mechanism of immune protection against nasal infection with *B. pertussis*. Since previous studies on *Streptococcus pneumonia*,^30^ *Staphylococcus aureus*^31,32^ and *Pseudomonas aeruginosa*^33^ had suggested that IL-17 plays a key role in protection against respiratory nasal infection with these pathogens, we focused on the role of this cytokine. However, we did not exclude a role for IFN-γ or Th1 cells, which we have previously demonstrated play a critical role in protection against lung infection with *B. pertussis*.^28^ Our data reveal that a high proportion of pathogen-specific T_RM_ cells recruited to the nasal tissue during *B. pertussis* infection secreted IL-17 and comparatively fewer secreted IFN-γ. Mice lacking IL-17 have significantly impaired ability to clear a primary infection with *B. pertussis*. Neutralization of IL-17 prior to re-challenge of convalescent mice completely prevented clearance of the bacteria from the nasal cavity. Defects in bacterial clearance from the nasal mucosae during primary or secondary infections of *Il17A*^−/−^ mice with *B. pertussis* were associated with significantly reduced recruitment of Siglec-F^+^ neutrophils. Finally, depletion of CD4 T cells or neutrophils impaired clearance of *B. pertussis* from the nasal cavity. Our study demonstrates that IL-17-mediated recruitment of neutrophils, especially Siglec-F^+^ neutrophils, is central to protection against nasal infection with *B. pertussis*.

## Results

### IL-17-secreting CD4 T cells and Siglec-F^+^ neutrophils are recruited to the nasal cavity during *B. pertussis* infection

**Fig. 1:**
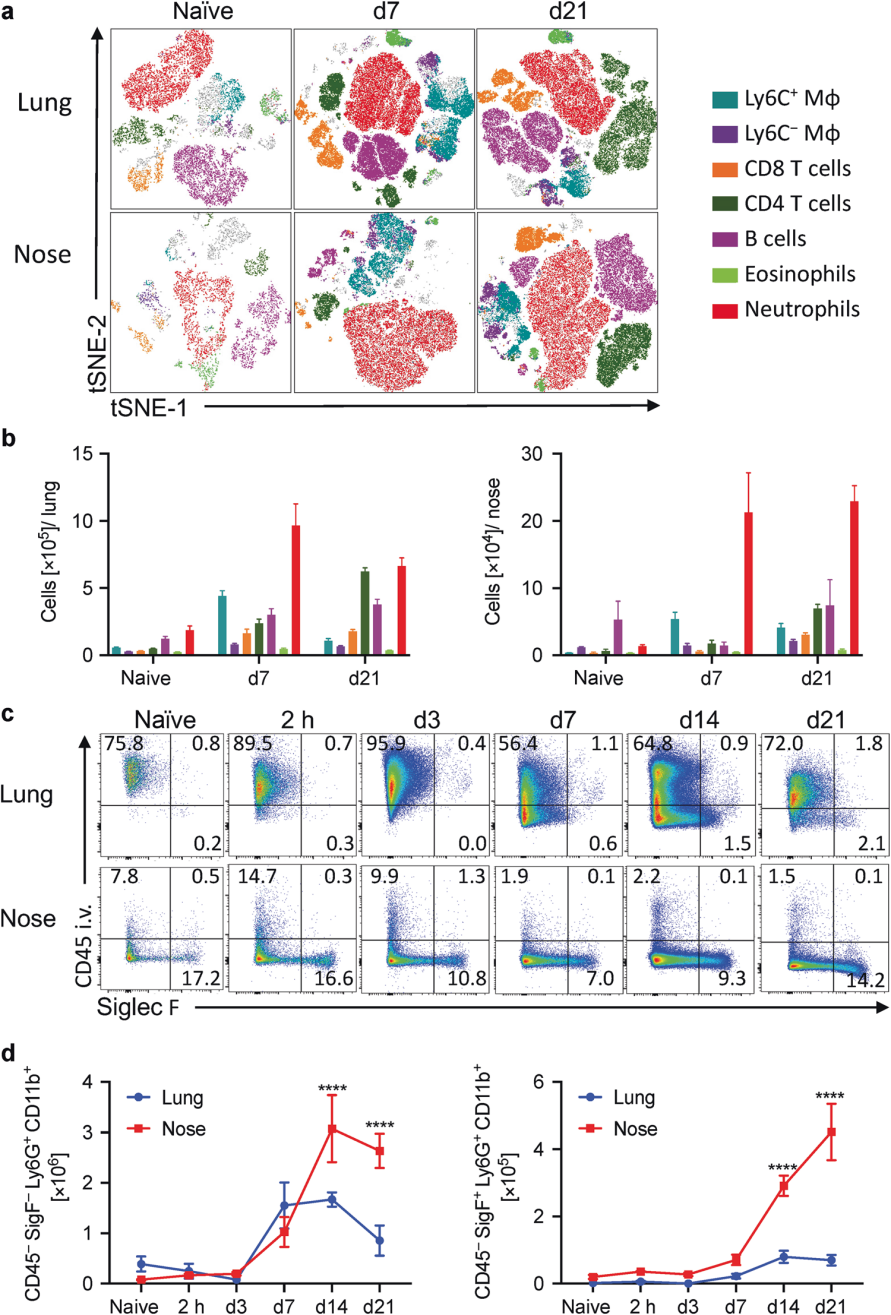
Siglec-F^+^ neutrophils are recruited to the nasal tissue during *B. pertussis* infection. C57BL/6 mice were aerosol infected with *B. pertussis*. At different time points, mice were injected i.v. with fluorochrome-labeled CD45 antibody and euthanized 10 min later. Cell suspensions were prepared from lung and nasal tissue and immune cells were analyzed by flow cytometry. **a** Representative tSNE plots for cells from lung and nasal tissue in naive mice and 7 or 21 days post infection. Neutrophils (Ly6G^+^Ly6C^+^), eosinophils (Siglec-F^+^ CD11c^−^), B cells (B220^+^MHCII^+^), CD4 T cells (CD3^+^CD4^+^), CD8 T cells (CD3^+^CD8^+^), Ly6C^−^ macrophages (F4/80^+^CD11b^+^Ly6C^−^), Ly6C^+^ macrophages (F4/80^+^CD11b^+^Ly6C^+^). **b** Absolute cell counts for immune cell populations in lung and nose. **c** Cells were pre-gated on total neutrophils (Ly6G^+^CD11b^+^), dot plots show the intravital CD45 stain on the *y*-axis and Siglec-F on the *x*-axis. **d** Absolute cell numbers of tissue-resident Siglec-F^−^ neutrophils (CD45 i.v.^−^ Siglec-F^−^Ly6G^+^CD11b^+^) and Siglec-F^+^ neutrophils in lung and nose. *n* = 4/group, mean ± SEM, statistical analysis: Two-way ANOVA followed by Sidak’s post-test, significances are indicated in comparison to naive mice of the same population, ****p < 0.0001.

Immunization with aP vaccines prevents severe disease in humans and infection in the lungs of mice but does not confer protective immunity against nasal infection with *B. pertussis* in mice or baboons.^13,14^ Nasal infection may facilitate asymptomatic transmission of *B. pertussis*, even in individuals immunized with aP vaccines. Therefore, it is important to understand the mechanism of protective immunity against infection in the nasal cavity, as well as in the lungs, in order to inform the design of improved pertussis vaccines. We have previously demonstrated that protection against lung infection in mice is mediated largely by IFN-γ-secreting Th1 cells, with a lesser role for IL-17-secreting Th17 cells.^28,34^ Here we examined the cellular immune responses in the nasal cavity of mice infected with *B. pertussis*. After aerosol challenge of mice with *B. pertussis*, there is significant infiltration of immune cells into the tissues of the nasal mucosae. Flow cytometry analysis displayed as tSNE plots showed substantial recruitment of neutrophils, CD4 T cells, B cells, and LyC6^+^ macrophages (monocyte derived) to the nasal tissue and lungs during *B. pertussis* infection (Fig. 1a, b).

Neutrophils were the dominant population of immune cells that infiltrated the nasal tissue during infection with *B. pertussis*, therefore, we examined this cell type in more detail. Neutrophils that express Sialic acid-binding, Ig-like lectin (Siglec)-F, have been found in the nasal mucosae of mice in a model of allergic rhinitis.^35^ Siglec-F^+^ neutrophils have an activated phenotype, produce reactive oxygen species (ROS) and have higher phagocytic activity than Siglec-F^−^ neutrophils.^35,36^ In order to quantify tissue-resident cells, we used a validated intravenous labeling approach,^37^ where mice are injected i.v. with fluorochrome-labeled anti-CD45 antibody 10 min before euthanasia and immune cells were prepared from nasal tissue and lung. This allowed us to discriminate circulating (CD45iv^+^) from tissue-resident (CD45iv^−^) cells in lungs or nasal tissue. We found significant expansion of Siglec-F^+^ and Siglec-F^−^ neutrophils in the nasal mucosae (Fig. 1c, d). Almost all Siglec-F^−^ and Siglec-F^+^ neutrophils in the nasal mucosae were tissue-resident, based on in vivo staining with anti-CD45 (Supplementary Fig. 1a, b). In the nasal tissue more than 90% of Siglec-F^−^ neutrophils and up to 95% of Siglec-F^+^ neutrophils were located deep in the nasal tissue, with less than 1% Siglec-F^+^ neutrophils showing intermediate staining with CD45. However, in lung tissue, only around 48% of Siglec-F^−^ neutrophils were tissue-resident, whereas more than 76% of Siglec-F^+^ neutrophils were tissue-resident (Supplementary Fig. 1a, b). These results show that the Siglec-F^+^ neutrophil subset is almost exclusively located within the tissue in nose as well as lung, while a big proportion of Siglec-F^−^ neutrophils are found in the circulating and intermediate populations in the lung, but not in the nose, highlighting organ-specific differences in neutrophil recruitment or distribution.

**Fig. 2:**
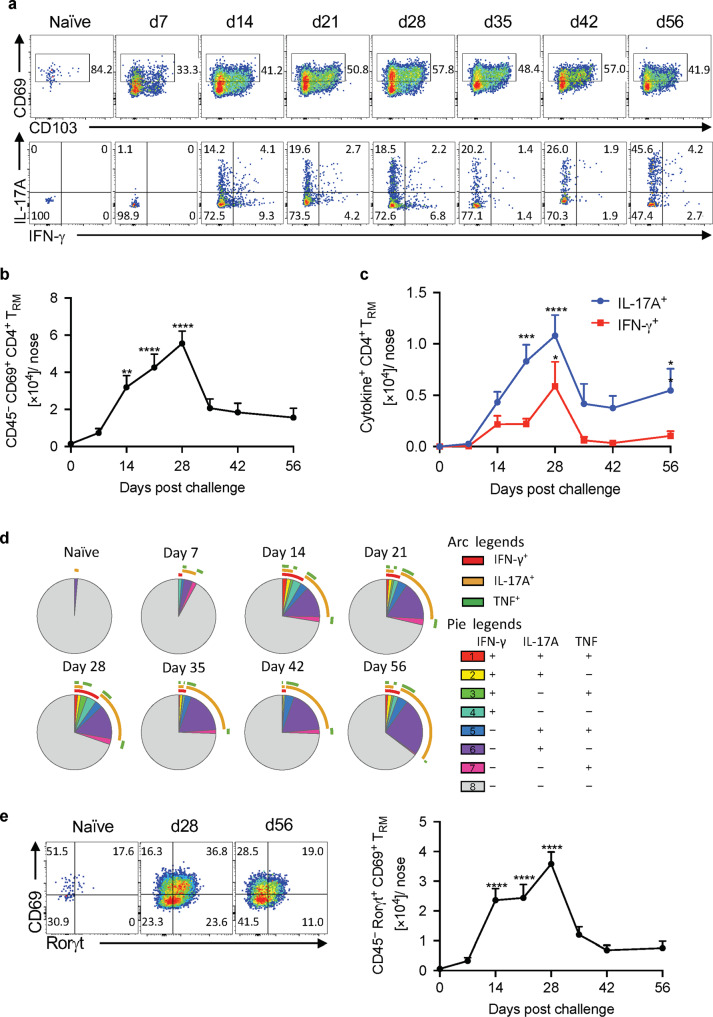
IL-17A-secreting CD4 T_RM_ cells accumulate in the nasal tissue during *B. pertussis* infection. C57BL/6 mice were aerosol infected with *B. pertussis*. At different time points, mice were injected i.v. with fluorochrome-labeled CD45 antibody and sacrificed 10 min later. Cell suspensions were prepared from nasal tissue and cells were stimulated with sBP and anti-CD49d/CD28 and analyzed by flow cytometry. **a** CD69 and CD103 expression on tissue-resident CD44^+^ CD4^+^ T cells and IL-17A and IFN-γ-production in the CD69^+^ sub-population. **b** Total number of CD4 T_RM_ (CD45 i.v.^−^ CD4^+^ CD44^+^ CD69^+^) in nasal tissue during *B. pertussis* infection. **c** Total number of *B. pertussis*-specific IL-17A- or IFN-γ-producing CD4 T_RM_ cells. **d** SPICE analysis of cytokine production in sBP-stimulated CD4 T_RM_. **e** Analysis of RORγT expression in CD4 T_RM_ cells in naive mice and 28 and 56 days post infection. Absolute numbers of tissue-resident RORγt^+^ CD69^+^ CD4 T cells. Statistical analysis: **b** One-way ANOVA followed by Dunnett’s post-test, significances are indicated in comparison to naive mice; **c** Two-way ANOVA followed by Sidak’s post-test, significances are indicated in comparison to naive mice of the same population; *p < 0.05, **p < 0.01, ***p < 0.001, ****p < 0.0001, *n* = 4/group, mean ± SEM.

Since neutrophil recruitment is enhanced by T cells, especially Th17 cells, we examined the recruitment of tissue-resident T cells, including *B. pertussis*-specific T_RM_ cells over the course of infection. Tissue-resident T cells were quantified using intravenous labeling with anti-CD45 to identify tissue-resident (CD45iv^-^) cells, together with staining for CD69 and CD103, markers expressed on some but not all T_RM_ cells.^38^ Cells in the CD45iv^-^ population, that are CD44^+^CD62L^−^ and express CD69, with or without CD103, are considered to be T_RM_ cells. The data revealed significant recruitment of CD69^+^ tissue-resident CD4 T cells into the nasal tissue, which peaked on day 28 (Fig. 2a, b). CD4 T_RM_ cells were also detected in the NALT of *B. pertussis* infected mice, however, B cells were the dominant population in this tissue (Supplementary Fig. 2)

We assessed the frequency of these nasal T_RM_ cells that were *B. pertussis*-specific and the cytokines they secreted in response to antigen stimulation. Nasal tissue cells were prepared from naive and infected mice and stimulated for 18 h with *B. pertussis* antigen. Brefeldin-A was added for the last 4 h of culture and cells were then stained for surface CD3 and CD4, CD44 and CD69 and intracellular IL-17, IFN-γ and TNF. The data revealed significant numbers of IL-17-secreting *B. pertussis*-specific tissue-resident CD4 T cells in the nasal tissue, which peaked on day 28 (Fig. 2c). Over 20% of all CD4 T_RM_ cells in the nasal tissue on day 28 of infection were IL-17-secreting *B. pertussis*-specific T_RM_ cells. The numbers declined after clearance of the bacteria but were still relatively high 56 days after challenge. IFN-γ-secreting *B. pertussis*-specific CD4 T_RM_ cells were also expanded in the nasal tissue of *B. pertussis*-infected mice, but the numbers were significantly lower than IL-17-secreting T_RM_ cells (Fig. 2c). SPICE analysis revealed that the dominant cytokine producing CD4 T cells in the nasal tissue were IL-17-secreting, with minor populations that secreted IL-17 and TNF or IL-17 and IFN-γ (Fig. 2d). In addition, staining of unstimulated T cells for the transcription factor RORγt which is essential for the differentiation of Th17 cells,^39^ revealed that over 36% of CD69^+^ T_RM_ cells from *B. pertussis*-infected mice expressed RORγt on day 28 post infection (Fig. 2e). IL-17- and IFN-γ-secreting *B. pertussis*-specific CD4 T_RM_ cells were also expanded in the lungs of *B. pertussis*-infected mice (Supplementary Fig. 3).

Our findings demonstrated that significant numbers of antigen-specific IL-17-producing CD4 T_RM_ cells and Siglec-F^+^ neutrophils accumulate in the nasal tissue during primary infection of mice with *B. pertussis*. Therefore, it is possible that local tissue-resident Th17 cells may be particularly important for recruitment of Siglec-F^+^ neutrophils to the respiratory tract, especially to the nasal mucosae of mice infected with *B. pertussis*.

### IL-17 is required for clearance of a primary infection of *B. pertussis* from the nasal cavity

**Fig. 3:**
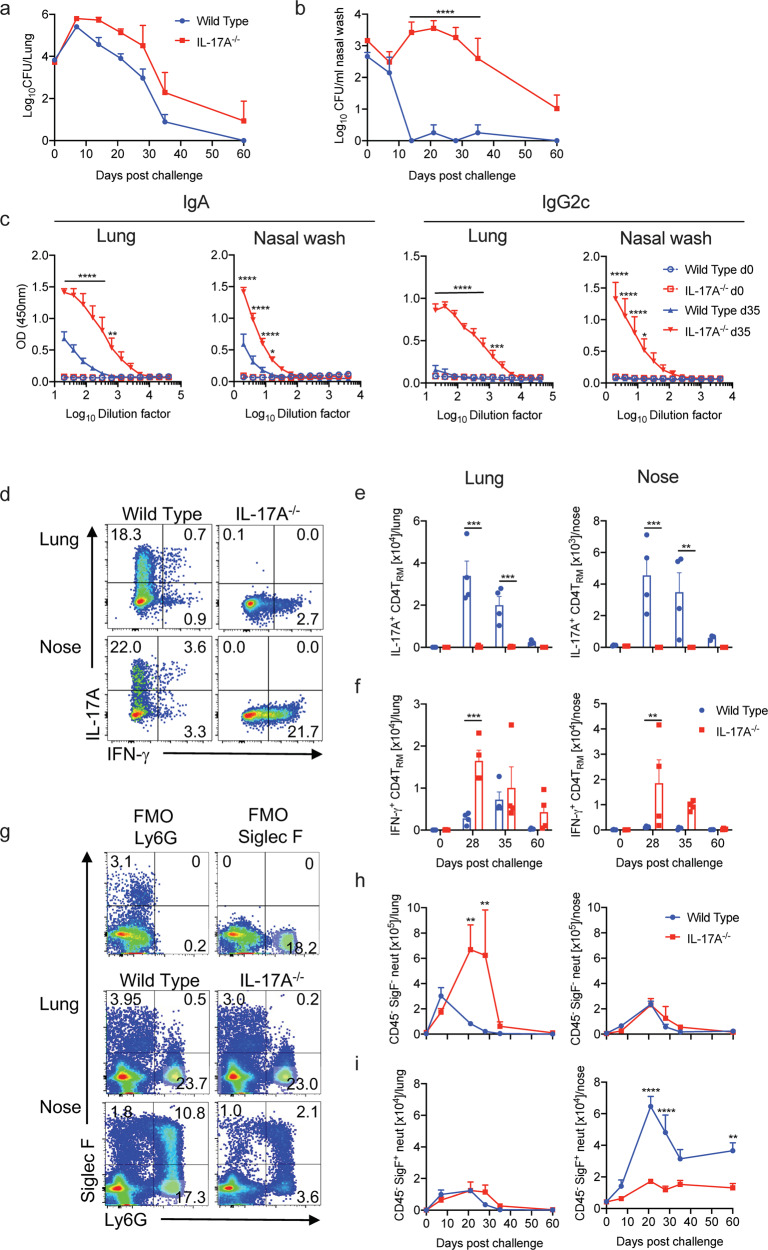
*Il17A*^−/−^ mice fail to clear *B. pertussis* infection in the nasal cavity. WT and *Il17A*^−/−^ mice were aerosol infected with *B. pertussis*. At different time points, mice were injected i.v. with fluorochrome-labeled CD45 antibody and euthanized 10 min later. Cell suspensions were prepared from lung and nasal tissue and cells were stimulated with sBP and CD49d/CD28 and analyzed by flow cytometry. CFU counts in lungs (**a**) and nasal washes (**b**) of WT and *Il17A*^−/−^ mice. **c** *B. pertussis*-specific IgA and IgG2c in nasal washes and lung homogenates. **d** Representative dot plots of IL-17A- and IFN-γ-production in CD4 T_RM_ cells in lung and nose of WT and *Il17A*^−/−^ on d 28 post infection. Total number of IL-17A (**e**) and IFN-γ (**f**) producing CD4 T_RM_ in lung and nose. **g** Representative dot plots of Ly6G and Siglec-F expression in lung and nose cell suspensions of WT and *Il17A*^−/−^ mice on d 28 post infection. Cell counts of tissue-resident Siglec-F^−^ (**h**) and Siglec-F^+^ (**i**) neutrophils in lungs and nasal tissue. Statistical analysis: **a**, **b**, **e**, **f**, **h**, **i** Two-way ANOVA followed by Sidak’s post-test, **c** Two-way ANOVA comparing WT and IL-17A^−/−^ d35 followed by Sidak’s post-test, *p < 0.05, **p < 0.01, ***p < 0.001, ****p < 0.0001, *n* = 4/group, mean ± SEM.

Having established that IL-17-secreting T_RM_ cells are expanded in the nasal tissue of mice during *B. pertussis*, we examined the role of IL-17 in clearance of infection from the nasal cavity, and for comparison purposes, we also examined the bacterial clearance from lungs. We had previously shown that *Il17A*^−/−^ mice have enhanced bacterial load in the lungs,^34^ and consistent with this, we found that the CFU counts were higher in the lungs of *Il17A*^*−/−*^ compared with wild type (WT) mice throughout the course of infection (Fig. 3a). However, nasal infection was dramatically more severe and prolonged in *Il17A*^−/−^ compared with WT mice. *Il17A*^−/−^ mice were still nasally infected within 60 days after *B. pertussis* challenge (Fig. 3b). We confirmed these data using co-housed WT and *Il17A*^−/−^ mice. The course of a primary infection was significantly exacerbated in *Il17A*^−/−^ compared with WT mice and this was most dramatic in the nasal mucosa (Supplementary Fig. 4).

Since IL-17 is known to be involved in IgA secretion,^40^ as well as neutrophil recruitment, and since murine IgG2c is involved in opsonization of bacteria by neutrophils and macrophages, we assessed antibody responses in *B. pertussis* infected *Il17A*^−/−^ compared with WT mice. Analysis of antibody responses on day 35 post challenge revealed significantly higher *B. pertussis*-specific IgA and IgG2c titers in nasal washes and lung homogenates of *Il17A*^−/−^ compared with WT mice (Fig. 3c). This may reflect the significantly higher bacterial load in those animals, and while it does not rule out a protective role for antibody, especially in opsonization of *B. pertussis*, it suggests that *B. pertussis-*specific IgA and IgG2c may not be essential for clearance of a primary infection with *B. pertussis*.

We also assessed the local T cell responses in the respiratory tract of *B. pertussis* infected *Il17A*^−/−^ and WT mice. Consistent with the data in Fig. 2, we found significant expansion of *B. pertussis*-specific IL-17-producing CD4 T_RM_ in lung and nasal tissue of WT mice infected with *B. pertussis*, and as expected, these were absent from *Il17A*^−/−^ mice (Fig. 3d, e). IFN-γ-producing T_RM_ cells were also expanded in the lungs 28 and 35 days post *B. pertussis* challenge of WT mice, but were very low or undetectable in the nasal tissue of the same mice (Fig. 3d, f). In contrast, there was significant expansion of IFN-γ-producing CD4 T_RM_ in the nasal tissue and lungs of *Il17A*^−/−^ mice (Fig. 3d, f). Taken together with the data showing significantly higher bacterial load in *Il17A*^−/−^ mice, this provides evidence that Th17 rather than Th1 cells are the critical T cell subset for protective immunity against a primary infection of the nasal mucosa with *B. pertussis*.

Finally, we examined neutrophil recruitment to the site of infection in *Il17A*^−/−^ and WT mice. The number of Siglec-F^+^ neutrophils in the lungs was similar in *Il17A*^*−/−*^ and WT mice after challenge with *B. pertussis* (Fig. 3g, i). However, the number of Siglec-F^−^ neutrophils was significantly higher in the lungs on days 21 and 28 after *B. pertussis* challenge of *Il17A*^−/−^ mice (Fig. 3g, h). In contrast, there was substantial recruitment of Siglec-F^+^ neutrophils to the nasal tissue of WT mice during the course of infection with *B. pertussis*, and this was reduced to background levels in *Il17A*^−/−^ mice (Fig. 3g, i). Taken together, our findings demonstrate that IL-17 plays a critical role in the clearance of a primary *B. pertussis* infection, especially from the nasal cavity, and that this may involve recruitment of neutrophils, especially Siglec-F^+^ neutrophils.

### Depletion of neutrophils impairs bacterial clearance from the nasal cavity following primary infection with *B. pertussis*

Our results so far have established that IL-17 plays a critical role in bacterial clearance from the nasal mucosae following primary infection with *B. pertussis* and that this was associated with recruitment of Siglec-F^+^ neutrophils to the nasal tissue. Furthermore, mice lacking IL-17 had impaired bacterial clearance and impaired recruitment of Siglec-F^+^ neutrophils into the nasal tissue. In order to confirm that neutrophils play a key role in IL-17-mediated protection against *B. pertussis*, we depleted neutrophils during primary infection with *B. pertussis*. Previous studies did not find a role for neutrophils in control of a primary infection of the lungs with *B. pertussis*.^41^ These studies were based on depletion using the anti-GR1 antibody RB6-8C5 which recognizes Ly6G, but also Ly6C expressed on other cell types.^42^ Here we followed the protocol established by Boivin et al.,^43^ where mice were treated daily with anti-Ly6G antibody (1A8, rat IgG2a) in combination with a secondary anti-rat mouse IgG2a antibody (MAR 18.5; ortholog to rat IgG2b) in order to switch the mechanism of depletion from opsonization to complement-mediated cell death.

**Fig. 4:**
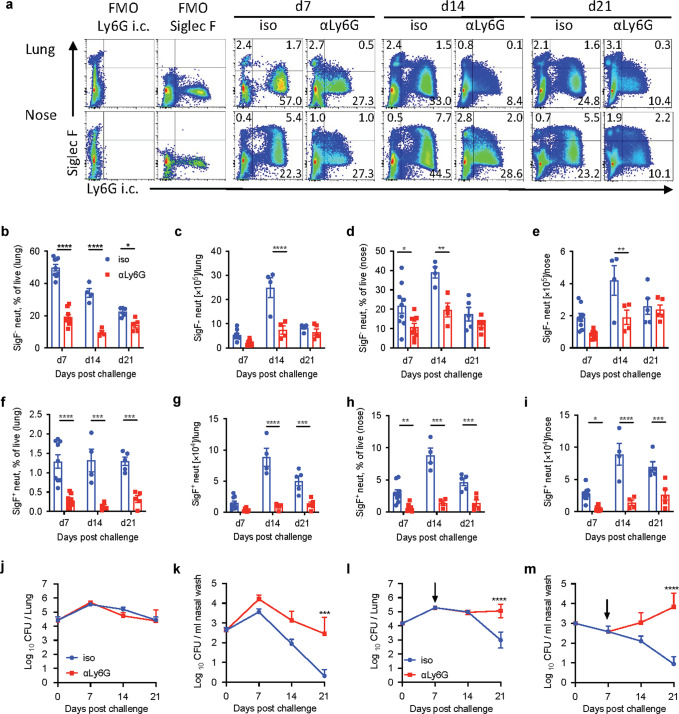
Depletion of neutrophils during primary infection with *B. pertussis* impairs bacterial clearance from nasal mucosae. C57BL/6 mice were treated with anti-Ly6G or isotype control antibody (iso) in combination with secondary antibody MAR 18.5 starting from d −1, and aerosol infected with *B. pertussis*. At 7, 14 and 21 days post infection, mice were injected i.v. with fluorochrome-labeled CD45 antibody and euthanized 10 min later. Cell suspensions were prepared from lung and nasal tissue and immune cells were analyzed by flow cytometry. **a** Representative dot plots showing the reduction of Siglec-F^+^ and Siglec-F^−^ neutrophils (intracellular Ly6G^+^) in anti-Ly6G treated mice compared with isotype control antibody treated mice 7, 14 and 21 days post infection. Percentage of Siglec-F^−^ neutrophils in lung (**b**) and nasal tissue (**c**). Absolute numbers of Siglec-F^−^ neutrophils in lung (**d**) and nasal tissue (**e**). Percentage of Siglec-F^+^ neutrophils in lung (**f**) and nasal tissue (**g)**. Absolute numbers of Siglec-F^+^ neutrophils in lung (**h**) and nasal tissue (**i**). CFU counts in lung (**j**) and nasal washes (**k**) of anti-Ly6G and isotype control treated mice. C57BL/6 mice were aerosol infected with *B. pertussis* and treated with anti-Ly6G or isotype control in combination with secondary antibody MAR 18.5 starting from d 7 post infection. CFU counts in lung (**l**) and nasal washes (**m**) of anti-Ly6G and isotype control antibody treated mice. Statistical analysis: Two-way ANOVA followed by Sidak’s post-test, *p < 0.05, **p < 0.01, ***p < 0.001, ****p < 0.0001. *n* = 4–8/group, pooled data from two independent experiments, mean ± SEM.

The surface epitope for the anti-Ly6G antibody 1A8 is masked in anti-Ly6G-treated animals, therefore we quantified neutrophils in lung and nasal tissue based on intracellular labeling for Ly6G.^43^ We found significantly reduced percentages and absolute numbers of Siglec-F^−^ neutrophils in the lungs and nasal tissue of mice treated with anti-Ly6G (Fig. 4a–e). However, the more consistent depletion across the course of infection was observed for Siglec-F^+^ neutrophils, with a significant reduction at 7, 14 and 21 days post *B. pertussis* challenge (Fig. 4a, f–i). Analysis of the effect of neutrophil depletion on infection with *B. pertussis* revealed that there was no difference in the bacterial load in the lungs of mice treated with anti-Ly6G compared with the isotype control antibody (Fig. 4j). In the nasal washes, however, CFU counts were higher on day 7 and 14 and significantly higher 21 days after challenge of mice treated with anti-Ly6G compared with the isotype control antibody (Fig. 4k). We also depleted neutrophils starting on day 7 and found that this constrained bacterial clearance from the lungs (Fig. 4l) and had a more dramatic effect on clearance of *B. pertussis* from the nasal cavity (Fig. 4m). The bacterial load in the nasal mucosa increased on day 14 and further on day 21 in anti-Ly6G-treated mice, when it was nearly 1000-fold higher than in mice treated with the isotype control antibody.

In order to consolidate the link between IL-17, produced primarily by *B. pertussis*-specific CD4 T_RM_ cells in the nasal cavity, and Siglec-F^+^ neutrophils and bacterial clearance, we depleted neutrophils in *Il17A*^−/−^ mice during primary infection with *B. pertussis*. The results demonstrated that treatment of *Il17A*^−/−^ mice with anti-Ly6G had no effect on the numbers of CFU in the lungs or nasal mucosa after *B. pertussis* challenge (Supplementary Fig. 5).

These data demonstrate that neutrophils, play an important role in the clearance of bacteria from the nasal cavity during primary infection with *B. pertussis*. Since the anti-Ly6G antibody was particularly effective at depleting Siglec-F^+^ neutrophils and since these cells are selectively reduced in *Il17A*^−/−^ mice, which have defective clearance of bacteria from the nasal mucosa, our findings point to a protective role for IL-17-induced Siglec-F^+^ neutrophils in protective immunity against infection of the nose with *B. pertussis*, but do not rule out a role for Siglec-F^−^ neutrophils.

### Depletion of CD4 T cells reduces protective immunity against primary infection and re-infection of the nasal mucosa

**Fig. 5:**
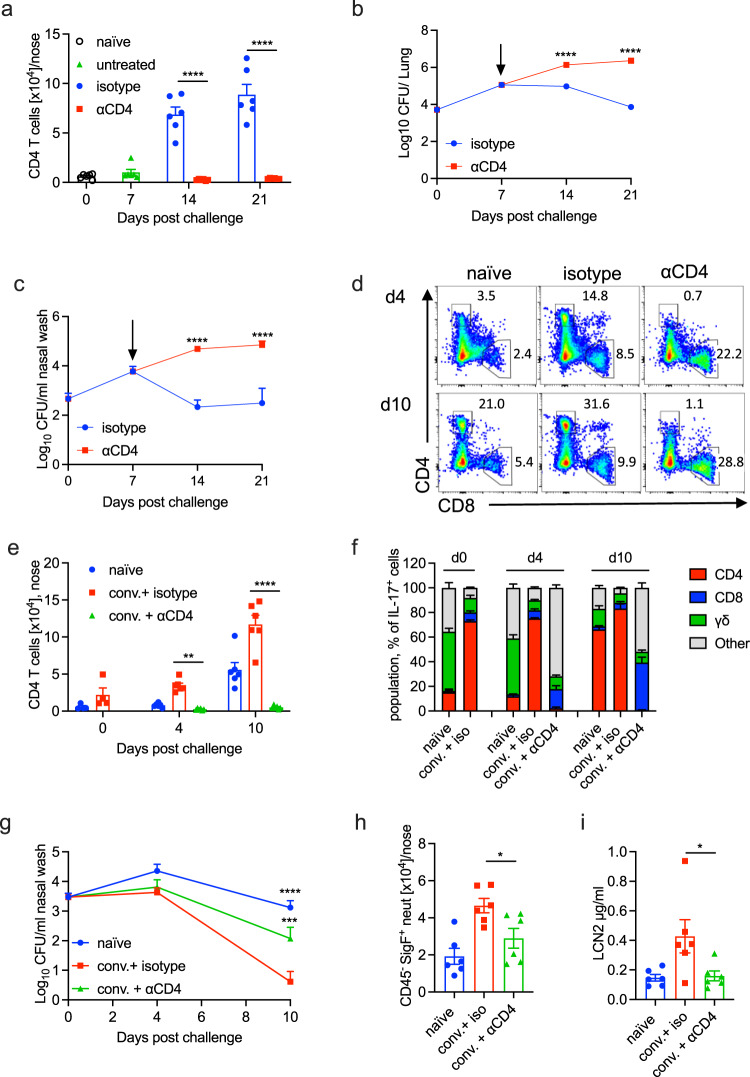
Depletion of CD4 T cells impairs clearance of *B. pertussis* from the nasal mucosa in primary infection and after challenge of convalescent mice. C57BL/6 mice were aerosol infected with *B. pertussis*. Beginning on day 7 post infection, mice were treated by i.p. and i.n administration of a neutralizing anti-CD4 antibody or an isotype control antibody. At 7, 14 and 21 days post infection, mice were injected i.v. with fluorochrome-labeled CD45 antibody and euthanized 10 min later. Cell suspensions were prepared from lung and nasal tissue and immune cells were analyzed by flow cytometry. CFU counts in lung (**a**) and nasal washes (**b**) of anti-CD4 or isotype control antibody treated mice. **c** Absolute counts of CD4 T cells in nasal tissue of anti-CD4 or control antibody treated mice. C57BL/6 mice were aerosol infected with *B. pertussis* and allowed to clear the infection. Convalescent mice were treated by i.p. and i.n administration of a neutralizing anti-CD4 antibody or an isotype control antibody from day −1 of re-challenge with *B. pertussis*. Nasal tissue cells were prepared 4 and 10 days after re-challenge or from control mice given a primary challenge on the same day. All mice were injected i.v. with fluorochrome-labeled CD45 antibody 10 min before euthanasia. Cell suspensions were prepared from nasal tissue, stimulated with PMA and ionomycin and immune cells were analyzed by flow cytometry. **d** Representative dots plot of nasal CD4 versus CD8 T cells pre-gated on CD3. **e** Absolute counts of CD4 T cells in nasal tissue of anti-CD4 or isotype control antibody treated mice. **f** Relative proportion of IL-17-secreting CD4 T cells, CD8 T cells, γδ T cells or other cell types in the nasal cavity on days 0, 4 or 10 after *B. pertussis* challenge of naive or convalescent mice. **g** CFU counts in the nasal wash of naive mice or convalescent mice treated with anti-CD4 or isotope control antibody. **h** Tissue-resident Siglec-F^+^ neutrophils in nasal tissue and **i** concentration of LCN2 in nasal wash 4 days post challenge. Statistical analysis: **a**, **b**, **c**, **e**, **g** Two-way ANOVA followed by Sidak’s post-test; **h**, **i** One-way ANOVA followed by Tukey’s post-test, *p < 0.05, **p < 0.01, ***p < 0.001, ****p < 0.0001. **d**–**i** Pooled data from two independent experiments, *n* = 6–10/group, mean ± SEM.

Our results so far have established a role for IL-17 and neutrophils in clearance of a primary infection with *B. pertussis*. Furthermore, we have shown that IL-17-secreting CD4 T_RM_ cells are expanded in the nasal tissue during primary infection with *B. pertussis*. These data point to a role for CD4 T_RM_ cells that secrete IL-17 in protective immunity in the nasal cavity. In order to provide more definitive evidence of a protective role for local CD4 T cells in clearance of a primary infection of *B. pertussis*, we used a CD4 T cell depletion approach,^44^ that depletes CD4 T cells from nasal tissue as well as the circulation. Treatment of mice with anti-CD4 by intranasal (i.n.) and intraperitoneal (i.p.) administration of the antibodies from day 7 of infection significantly reduced the numbers of CD4 T cells in the nasal tissue during infection with *B. pertussis* (Fig. 5a). Depletion of CD4 T cells resulted in significantly impaired clearance of *B. pertussis* from the lungs (Fig. 5b) and nasal mucosa (Fig. 5c), but the most dramatic effects was seen in the nose.

We next examined the role of CD4 T cells in protective immunity against re-infection of convalescent mice. Mice that had been infected with *B. pertussis* were left to convalesce for 1 year and then re-challenged with *B. pertussis*. Convalescent mice had significantly lower bacterial load in the nasal cavity 7 days after *B. pertussis* challenge when compared with challenge of naive mice (Supplementary Fig. 6a). The enhanced ability to clear the infection in long-term convalescent mice was reflected in a significantly higher number of *B. pertussis* specific IL-17-producing CD4^+^ T_RM_ cells in the nasal cavity 7 days after challenge (Supplementary Fig. 6b, d). IFN-γ-producing CD4^+^ T_RM_ cells were also expanded in the nasal cavity after re-challenge of convalescent mice (Supplementary Fig. 6c, d). However, there were more than 5-fold more IL-17- compared with IFN-γ-producing CD4^+^ T_RM_ cells.

We next examined the role for CD4 T_RM_ cells in adaptive immunity against re-infection by depleting CD4 T cells 5 and 1 days prior to and every 4 days after re-challenge of convalescent mice with *B. pertussis*. Treatment of mice with anti-CD4 antibodies, by i.n. and i.p. administration, significantly reduced the numbers of CD4 T cells in the nasal tissue during re-infection with *B. pertussis* (Fig. 5d, e). Furthermore, the IL-17-secreting T_RM_ cells in the nasal tissue were reduced to background 4 and 10 days after *B. pertussis* challenge in the anti-CD4-depleted mice (Supplementary Fig. 6e, f).

We examined the cellular sources of IL-17 in the nasal cavity of *B. pertussis* infected mice and revealed that CD4 cells were the dominant source of IL-17 by day 10 of primary infection and in convalescent mice (Fig. 5f). The proportion of IL-17-secreting CD4 T cells increased further after re-challenge of convalescent mice and this was reduced to background in mice treated with anti-CD4 antibodies. γδ T cells were a significant source of IL-17 in the nasal cavity in naive mice and 4 days after primary *B. pertussis* challenge (Fig. 5f). CD8 T cells and other un-identified cells, possibly NKT or ILCs, were a minor source of IL-17 during primary or secondary infection with *B. pertussis* and the proportion of these was enhanced in anti-CD4 treated mice (Fig. 5f).

In order to assess the impact of depleting CD4 T cells from the nasal cavity on the course of infection, we assessed CFU counts in the nose. We found a significantly higher bacterial load in the nasal mucosa 10 days after *B. pertussis* challenge of anti-CD4 depleted mice (Fig. 5g). The number of CFU in the nasal mucosa of anti-CD4 treatment convalescent mice was close to that of un-treated naive mice 10 days after *B. pertussis* challenge. This was associated with a reduction in infiltrating Siglec-F^+^ neutrophils and a reduction in production of the antimicrobial peptide LCN2 (Fig. 5h, i). Collectively, these findings suggest that IL-17, predominantly, though not exclusively, produced by CD4 T_RM_ cells in the nasal tissues, plays a key role in protective immunity against *B. pertussis* infection and reinfection of the nasal mucosa.

### The role of IL-17 in protective adaptive immunity to *B. pertussis*

**Fig. 6:**
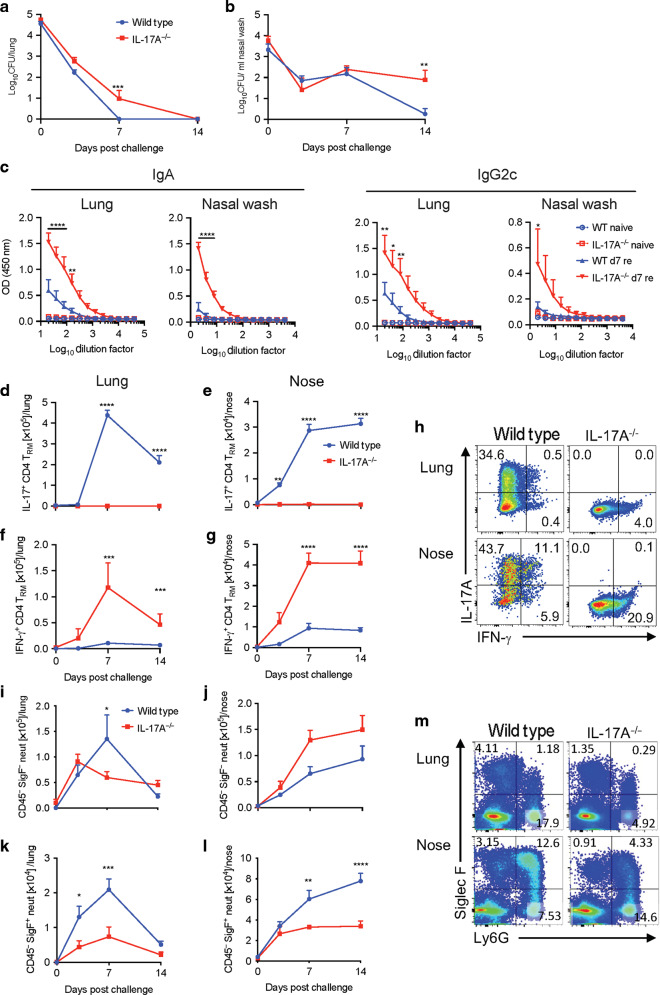
The role of IL-17 in protective acquired immunity to *B. pertussis*. WT and *Il17A*^−/−^ mice were aerosol infected with *B. pertussis* and left for 6 months to clear the infection, and then re-infected. Prior to re-infection and 3, 7 and 14 days post challenge, mice were injected i.v. with fluorochrome-labeled CD45 antibody and euthanized 10 min later. Cell suspensions were prepared from lung and nasal tissue and immune cells were analyzed by flow cytometry. CFU counts in lungs (**a**) and nasal wash (**b**) of re-infected WT and *Il17A*^−/−^ mice. **c** *B. pertussis*-specific IgA and IgG2c titers in nasal washes and lung homogenates 7 days post re-challenge. IL-17A-producing CD4 T_RM_ cells in lungs (**d**) and nasal tissue (**e**), and IFN-γ-producing CD4 T_RM_ in lungs (**f**) and nasal tissue (**g**). **h** Representative dot plots showing IL-17A and IFN-γ production in CD4 T_RM_ cells on day 7 post re-challenge. Siglec-F^−^ neutrophils in lung (**i)** and nasal tissue (**j**), and Siglec-F^+^ neutrophils in lung (**k**) and nasal tissue (**l**) during re-infection. **m** Representative dot plots showing Ly6G and Siglec-F expression on live cells in lungs and nasal tissue on day 7 of re-challenge. Statistical analysis: Two-way ANOVA followed by Sidak’s post-test comparing WT and IL-17A^−/−^ (**a**, **b**, **d**–**g**, **i**–**l**) or WT d7 re and IL-17A^−/−^ d7 re (**c**) *p < 0.05, **p < 0.01, ***p < 0.001, ****p < 0.0001. *n* = 4/group, mean ± SEM.

Having shown that IL-17-producing T_RM_ cells expand in the nasal tissue after re-challenge of convalescent mice and that these cells play a key role in clearance of bacteria, we examined the role of IL-17 in acquired protective immunity induced by natural infection. *Il17A*^−/−^ and WT mice were aerosol challenged with *B. pertussis* left for 6 months (when they cleared the infection; data not shown) and then re-challenged by aerosol with *B. pertussis*. Convalescent WT mice cleared the re-infection from the lungs by day 7 and from the nasal cavity by day 14. The CFU counts in the lungs were significantly higher in the lungs of *Il17A*^−/−^ compared with WT mice on day 7 and significantly higher in the nasal cavity 14 days after re-challenge of *Il17A*^−/−^ compared with WT mice (Fig. 6a, b).

Since mucosal IgA and serum IgG have been implicated in protective immunity against *B. pertussis*,^45,46^ we assessed antibody responses in *Il17A*^*−/−*^ and WT mice. IgA production after re-challenge of convalescent mice was higher in the lungs and nasal washes of *Il17A*^−/−^ compared with WT mice (Fig. 6c). Furthermore, IgG2c titers showed significantly higher titers in lungs and nasal washes of *Il17A*^*−/−*^ compared with WT mice (Fig. 6c). Although antibody responses were generally stronger after secondary compared with primary infection, our findings suggest that antibodies alone may not mediate acquired immunity induced by the previous infection with *B. pertussis* in the nasal mucosae.

Analysis of the T cell response after re-challenge of convalescent mice showed high numbers of *B. pertussis*-specific IL-17-secreting and lower numbers of IFN-γ producing T_RM_ cells in the nasal tissue and lungs of WT mice. While the IL-17-secreting T_RM_ cells were, as expected, absent in the *Il17A*^−/−^ mice, the number of IFN-γ-producing T_RM_ were dramatically enhanced in the lung and nasal tissue of *Il17A*^−/−^ mice (Fig. 6d–h). This is consistent with a more critical role for IL-17 than IFN-γ in acquired protective immunity in the nasal mucosae.

Assessment of neutrophils in the lung revealed that the substantial recruitment of Siglec-F^−^ and Siglec-F^+^ neutrophils 3–14 days after *B. pertussis* challenge of convalescent WT mice was significantly reduced in *Il17A*^−/−^ mice (Fig. 6i, k, m). In nasal tissue, the recruitment of Siglec-F^+^, but not Siglec-F^−^, neutrophils was reduced in *Il17A*^−/−^ mice after re-challenge with *B. pertussis* (Fig. 6j, l, m).

**Fig. 7:**
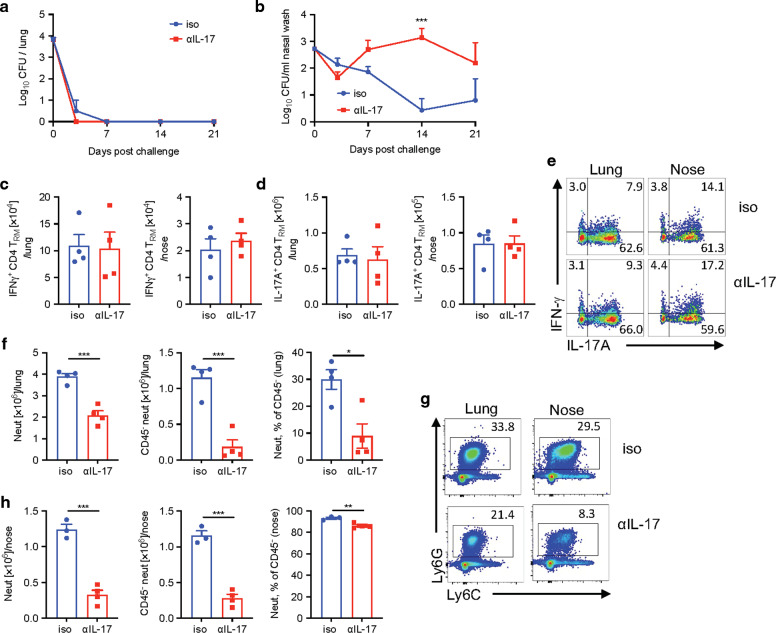
Depletion of IL-17 impairs clearance from the nasal cavity following re-challenge of convalescent mice. C57BL/6 mice were aerosol infected with *B. pertussis* and allowed to clear the infection. 3 months later, convalescent mice were re-challenged with *B. pertussis.* Mice were treated with anti-IL-17A or isotype control from one day before infection until the end of the experiment. Prior to infection and 7 days post challenge, mice were injected i.v. with fluorochrome-labeled CD45 antibody and euthanized 10 min later. Cell suspensions were prepared from lung and nasal tissue and immune cells were analyzed by flow cytometry. CFU counts in lungs (**a**) and nasal washes (**b**) of anti-IL-17A treated re-infected convalescent mice. IFN-γ- (**c**) and IL-17A (**d**) -producing CD4 T_RM_ cells in lungs and nasal tissue. **e** Representative dot plots of IL-17A- or IFN-γ-producing CD4 T_RM_ cells on day 7 post re-challenge of anti-IL-17A or isotype treated convalescent mice. **f** Absolute numbers of neutrophils, CD45 i.v.^−^ tissue-resident neutrophils, and % of CD45 i.v.^−^ cells in total neutrophils in the lungs. **g** Representative dot plots of total neutrophils (Ly6C^+^ Ly6G^+^) in lungs and noses on day 7 post re-challenge of anti-IL-17A treated convalescent mice. **h** Absolute numbers of neutrophils, CD45 i.v.^−^ tissue-resident neutrophils, and % of CD45 i.v.^−^ cells in total neutrophils in the nasal tissue. Statistical analysis: **a**, **b** Two-way ANOVA followed by Sidak’s post-test, ***p < 0.001; **c**, **d**, **f**, **h** unpaired, two-tailed *t*-test, *p < 0.05, **p < 0.01, ***p < 0.001; *n* = 4/group, mean ± SEM.

These data demonstrate that IL-17 plays a key role in natural immunity against re-infection of the nasal cavity and that this may be mediated by recruitment of Siglec-F^+^ neutrophils. However, one of the limitations of using cytokine-deficient mice to assess the mechanism of immunity against re-infection is that the primary infection was much more severe and, therefore, the antigen load was higher in *Il17A*^−/−^ mice and this may complicate conclusions on the role of IL-17 in protection against a secondary infection. Therefore, in order to separate the possible influence of IL-17 deficiency during the primary infection from the role of IL-17 in clearance of infection from convalescent mice, we used an anti-IL-17 antibody that we had previously shown to neutralize IL-17 in vivo in an IL-17-mediated autoimmune disease model.^47^ We examined the effect of blocking IL-17 on the course of re-infection with *B. pertussis* by administering the anti-IL-17 antibody the day before and every 3 days after *B. pertussis* challenge of convalescent WT mice. The results revealed that anti-IL-17 treatment had little effect on the rapid clearance of bacteria from the lungs following re-challenge of convalescent mice (Fig. 7a). In contrast, neutralization of IL-17 dramatically reduced the rate of bacterial clearance from the nasal mucosae of mice re-challenged with *B. pertussis*; the CFU counts remained high in the nasal cavity of anti-IL-17 treated mice, as late as 21 days post re-challenge (Fig. 7b).

In contrast, there were comparable numbers of IL-17- or IFN-γ-producing CD4 T_RM_ cells in lungs and nasal tissues (Fig. 7c–e) of convalescent mice treated with anti-IL-17 or an isotype control anybody immediately before and after re-challenge with *B. pertussis*. This demonstrates that IL-17 is not required for the expansion and function of T_RM_ cells and that depletion of already secreted IL-17 does not increase IL-17-production by T_RM_ cells by negative feedback. The findings suggest that once the Th17 cells have expanded in the lungs or nasal tissue during primary infection, unlike in *Il17A*^−/−^ mice, anti-IL-17 treatment before and after re-challenge with *B. pertussis* does not affect the frequency of the IL-17- relative to IFN-γ-secreting T cells in the respiratory tissue. However, the function of Th17-type T_RM_ cells or at least their signature cytokine IL-17 was inhibited. Evidence in support of this conclusion was provided by data showing a significant effect of anti-IL-17 treatment on neutrophil recruitment into respiratory tissue. The total number of neutrophils was significantly reduced in lungs and nasal tissues in anti-IL-17 treated mice (Fig. 7f–h). In vivo CD45 staining revealed that the frequency of tissue-resident neutrophils in the lungs was reduced from around 30% to 9% in anti-IL-17 treated mice (Fig. 7f). In the nasal tissue, most neutrophils are tissue-resident, but the frequency was significantly reduced in anti-IL-17 treated mice (Fig. 7h).

Taken together with the data from the *Il17A*^−/−^ mice (Fig. 6), our findings suggest IL-17 plays an essential role in natural immunity induced by the previous infection in the nasal mucosae, and that this may be mediated by recruitment of neutrophils, especially Siglec-F^+^ neutrophils.

### Depletion of neutrophils in convalescent mice during re-challenge delays bacterial clearance in the nasal cavity

**Fig. 8:**
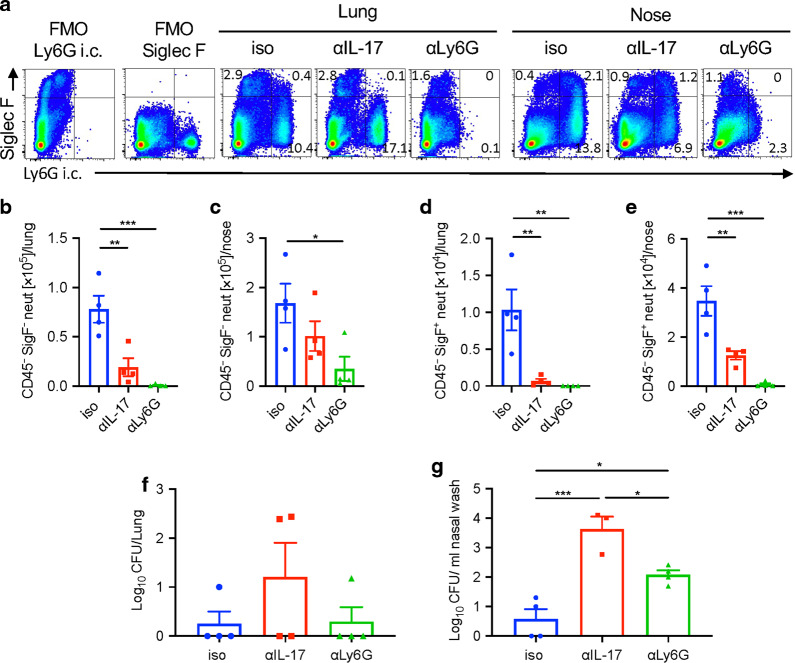
Depletion of neutrophils during re-challenge impairs clearance in the nose of convalescent mice. C57BL/6 mice were aerosol infected with *B. pertussis* and allowed to clear the infection. 4.5 months later, convalescent mice were re-challenged with *B*. *pertussis*. Mice were treated with anti-IL-17A or anti-Ly6G and 2nd antibody or isotype control from one day before infection until the end of the experiment. 7 days post challenge, mice were injected i.v. with fluorochrome-labeled CD45 antibody and euthanized 10 min later. Cell suspensions were prepared from lung and nasal tissue and immune cells were analyzed by flow cytometry. **a** Representative dot plots showing expression of Ly6G (intracellular) and Siglec-F on live cells in lungs and nasal tissue on day 7 post re-challenge. Absolute numbers of Siglec-F^−^ neutrophils in lung (**b**) or nasal tissue (**c**). Absolute numbers of Siglec-F^+^ neutrophils in lung (**d**) or nasal tissue (**e**). CFU counts in lungs (**f**) and nasal washes (**g**) on day 7 post re-challenge. Statistical analysis: One-way ANOVA followed by Tukey’s post-test, *p < 0.05, **p < 0.01, ***p < 0.001; *n* = 4/group, mean ± SEM.

Our studies on convalescent *Il17A*^−/−^ mice or on WT mice treated with anti-IL-17 prior to and after re-challenge with *B. pertussis* demonstrated that IL-17 played a key role in adaptive natural immunity against infection in the nasal mucosae. This was associated with a reduction in neutrophils, especially Siglec-F^+^ neutrophils in the nasal cavity. In order to provide more definitive evidence that the protective function of IL-17 was mediated through neutrophil recruitment, we depleted neutrophils using anti-Ly6G antibody (1A8, rat IgG2a) in combination with a secondary anti-rat mouse IgG2a antibody, as described above. We directly compared the effect of neutrophil depletion with the effect of neutralizing IL-17 in the clearance of infection in convalescent mice re-challenged with *B. pertussis*. Treatment with anti-Ly6G or neutralization of IL-17 significantly reduced the numbers of Siglec-F^+^ neutrophils in lungs and the nasal tissue (Fig. 8a, d, e), but only anti-Ly6G treatment significantly reduced the number of Siglec-F^-^ neutrophils in nasal tissue (Fig. 8a–c).

Assessment of CFU counts in the respiratory tract revealed that mice that had previously been infected 4.5 months earlier had effectively cleared the infection from the lungs 7 days after re-challenge. We found a small but not significant reduction in bacterial clearance from the lungs in anti-IL-17 treated mice, while anti-Ly6G treatment had little effect on the CFU counts in the lungs 7 days post-re-challenge (Fig. 8f). However, neutralization of IL-17 or depletion of neutrophils significantly impaired bacterial clearance from the nasal mucosae; CFU counts were significantly higher in nasal washes of anti-IL-17 or anti-Ly6G treated mice compared with those treated with the isotype control antibody (Fig. 8g). The effect of neutralizing IL-17 was somewhat stronger than depletion of neutrophils, suggesting that the protective effect of IL-17 is mediated at least in part by recruitment of neutrophils.

### IL-17 induces chemokines and cytokines necessary for neutrophil recruitment

**Fig. 9:**
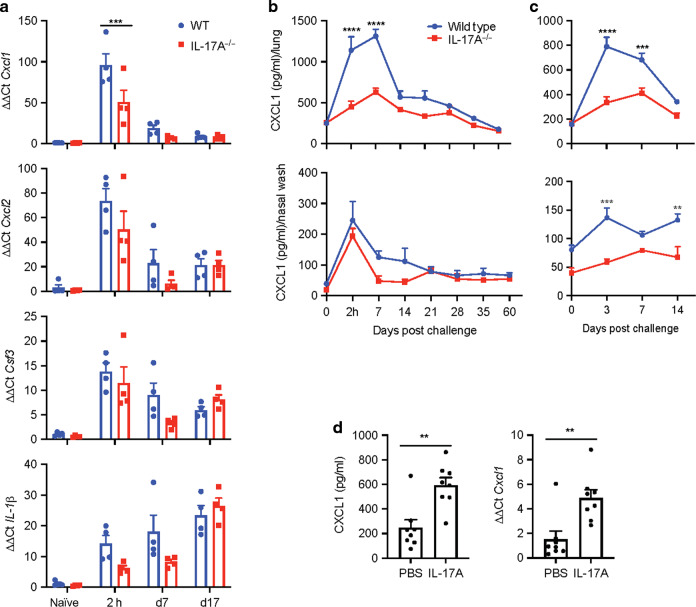
IL-17 induces chemokines and cytokines necessary for neutrophil recruitment. WT and *Il17A*^−/−^ mice were aerosol infected with *B. pertussis*. Prior to and 2 h, 7 and 17 d post challenge mice were sacrificed, and mRNA was extracted from nasal tissue for RT-PCR analysis. **a** Gene expression of *Cxcl1, Cxcl2, Cfs3* and *IL-1β* in nasal tissue of WT and *Il17A*^−/−^ mice was determined by RT-PCR and normalized to 18S RNA. **b** WT and *Il17A*^−/−^ mice were aerosol infected with *B. pertussis*. Up to day 60 post challenge nasal washes and lung homogenates were prepared and protein concentration of CXCL1 was determined by ELISA. **c** Convalescent WT and *Il17A*^−/−^ were aerosol infected with *B. pertussis*. On day 3, 7 and 14 post re-challenge nasal washes and lung homogenates were prepared and protein concentration of CXCL1 was determined by ELISA. **d** C57BL/6 mice were treated intranasally with IL-17A and euthanized 2 h later. CXCL1 concentration in nasal washes was determined by ELISA and *Cxcl1* gene expression was analyzed by RT-PCR and normalized to 18S RNA. Statistical analysis: Two-way ANOVA followed by Sidak’s post-test, **d** unpaired, two-tailed *t*-test, *p < 0.05, **p < 0.01, ***p < 0.001; **a**–**c** *n* = 4/ group; **d** *n* = 8/group, pooled data from two independent experiments, mean ± SEM.

IL-17 is known to promote neutrophil recruitment through induction of the chemokines CXCL1 and CXCL2, as well as GCSF, and pro-inflammatory cytokine IL-1β.^47,48^ We found that gene expression for *Cxcl1*, *Cxcl2* and *Csf3* and *Il1β* was significantly enhanced during infection with *B. pertussis* as early as 2 h post challenge (Fig. 9a). However, expression of *Cxcl1* was significantly lower in *Il17A*^−/−^ compared with WT mice. Expression of *Cxcl2, Csf3* and *Il1β* was also lower, though not significantly in *Il17A*^−/−^ mice.

Analysis of CXCL1 protein expression in lung homogenates and nasal washes showed rapid production of this chemokines within 2 h of *B. pertussis* challenge, which increased further over the first week of infection. The concentration of CXCL1 was significantly reduced in the lungs and reduced in the nasal cavity, though not significantly, in *Il17A*^−/−^ mice (Fig. 9b). We also found significantly reduced CXCL1 protein concentration in nasal washes and lungs of *Il17A*^−/−^ compared with WT mice following re-challenge of convalescent mice (Fig. 9c).

In order to confirm the role of IL-17 in the induction of CXCL1 in nasal tissue, naive WT mice were treated i.n. with IL-17. We found significantly increased concentrations of CXCL1 in the nasal washes and significantly increased expression of *Cxcl1* in nasal tissue 2 h after i.n. administration of IL-17, confirming the role of IL-17 in its induction in the nasal mucosae (Fig. 9d). Our findings suggest that IL-17 mediates neutrophil recruitment through induction of chemokines, especially CXCL1, in the respiratory tract soon after infection with *B. pertussis*.

### Mechanism of protection by Siglec-F^+^ neutrophils in the nasal mucosae

Our findings have demonstrated that IL-17 mediated recruitment of neutrophils plays a key role in protective immunity against nasal infection following primary *B. pertussis* challenge and in preventing re-infection in convalescent mice, and that Siglec-F^+^ neutrophils may play a key protective role, especially in the nasal mucosa. Therefore, we asked whether the recruitment of these cells was unique to the nasal tissue and what was different about these cells compared with conventional Siglec-F^−^ neutrophils.

**Fig. 10:**
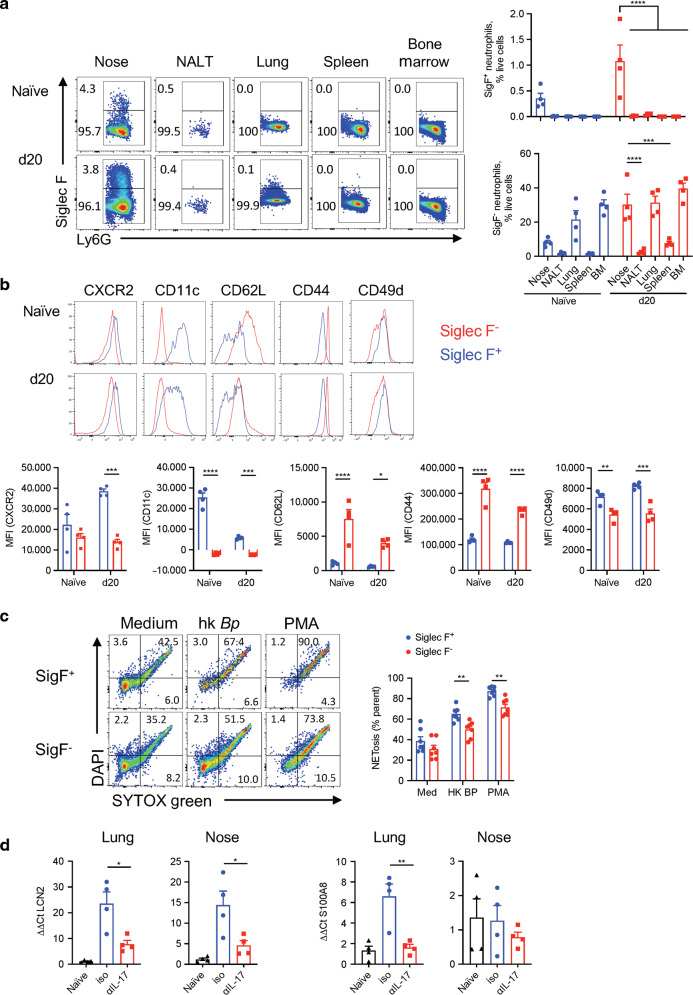
Siglec-F^+^ neutrophils have an activated phenotype and high NET activity. C57BL/6 mice were aerosol infected with *B. pertussis* and euthanized on 20 d post challenge. Cell suspensions were prepared from several tissues and immune cells were analyzed by flow cytometry. **a** Representative dot plots showing Siglec-F^+^ expression on CD11b^+^Ly6G^+^ pre-gated cells and percentages of Siglec-F^+^ and Siglec-F^−^ neutrophils in naive mice and on day 20 post challenge in nasal tissue, NALT, lung, spleen, and bone marrow. **b** Histograms and MFIs showing the expression of the surface markers CXCR2, CD11c, CD62L, CD44, and CD49d on Siglec-F^+^ and Siglec-F^−^ neutrophils in nasal tissue of naive mice and mice 20 d post challenge with *B. pertussis*. **c** Single cell suspension was prepared from nasal tissue of *B. pertussis* infected mice up to 1-month post challenge, was stimulated with heat-killed *B. pertussis* or PMA and then stained with the intracellular DNA dye (DAPI) and the cell-impermeant DNA dye (SYTOXgreen). Percentage of cells undergoing NETosis (double positive for DAPI and SYTOXgreen). **d** mRNA was prepared on day 6 post re-challenge from lungs and nasal tissue of convalescent mice treated with anti-IL-17A or isotype. Gene expression of *Lcn2* and *S100a8* was determined by RT-PCR and normalized against 18S RNA. Statistical analysis: **a** Two-way ANOVA followed by Tukey’s post-test, **b**, **c** Two-way ANOVA followed by Sidak’s post-test, **d** One-way ANOVA followed by Tukey’s post-test, *p < 0.05, **p < 0.01, ***p < 0.001; **a**, **b**, **d** *n* = 4/ group, **c** *n* = 7/group, pooled from two independent experiments, mean ± SEM.

A comparison of Siglec-F^+^ and Siglec-F^−^ neutrophils in different tissues revealed that Siglec-F^+^ were exclusive to nasal tissue in naive mice, and were also expanded in the lungs of *B. pertussis* infected animals, but the frequency was comparatively higher in the nasal tissue (Fig. 10a). In contrast, conventional Siglec-F^-^ neutrophils were found in the bone marrow, lungs and nasal tissue of naive mice and were also expanded in the spleen of *B. pertussis* infected mice (Fig. 10a).

Analysis of neutrophil surface marker expression revealed that CD11c, which is expressed on neutrophils during inflammation and sepsis, CD49d, the integrin α subunit of VLA-4, which is involved in migration of leukocytes, and CXCR2 the receptor for CXCL1, were enhanced on Siglec-F^+^ compared with Siglec-F^−^ neutrophils (Fig. 10b). In contrast, expression of CD62L and CD44 were lower on Siglec-F^+^ neutrophils (Fig. 10b). These findings are consistent with pro-inflammatory activity for Siglec-F^+^ neutrophils.

Neutrophils are known to mediate their function against bacterial infection in a number of ways, including antibody-mediated opsonization, neutrophil extracellular traps (NET) formation (NETosis) or through the production of antimicrobial peptides (AMPs).^49^ Although neutrophils can phagocytose and kill *B. pertussis* via antibody and complement and we do not rule this out, our data point to alternative mechanisms. We found that Siglec-F^+^ neutrophils had higher NETosis than conventional Siglec-F^−^ neutrophils and this was further enhanced by treatment with heat-killed *B. pertussis* or phorbol 12-myristate 13-acetate (PMA) (Fig. 10c).

IL-17 is known to induce the production of AMPs. While AMPs are primarily produced by epithelial cells, they can also be produced by neutrophils.^50^ LCN2 (also called NGAL, neutrophil gelatinase-associated lipocalin) is stored in neutrophil granules to be released upon activation.^51^ We found that expression of the AMP S100A8 was enhanced in respiratory tissue following re-challenge of convalescent mice and this was reduced by treatment with anti-IL-17 (Fig. 10d). Expression of the AMP *Lcn2* was also enhanced in the lungs and nasal tissue of convalescent mice following re-infection with *B. pertussis* and treatment with anti-IL-17 significantly reduced expression in both tissues (Fig. 10d). These data suggest that induction of AMP production may contribute to IL-17-induced protection against *B. pertussis* and this may be mediated via enhancement of neutrophil recruitment by IL-17. Collectively our findings suggest that IL-17 plays a critical role in protective immunity against *B. pertussis* infection and re-infection of the nasal mucosa and this is mediated to a significant extent by recruitment of neutrophils, especially Siglec-F^+^ neutrophils that are pro-inflammatory, with high NETosis activity and a potential source of protective AMPs.

## Discussion

The significant new findings of this study are that IL-17 secreted by CD4 T_RM_ cells plays a critical role in nasal clearance of *B. pertussis* following primary infection and is central to the mechanism of acquired immunity against secondary infection by promoting recruitment of neutrophils, especially Siglec-F^+^ neutrophils, to the nasal mucosae. We found that IL-17-producing pathogen-specific CD4 T_RM_ cells accumulate in the nasal tissue during infection with *B. pertussis* and these T_RM_ cells expanded locally following re-infection with *B. pertussis*. Depletion of CD4 T_RM_ cells significantly impaired clearance of a primary or secondary infection of the nose with *B. pertussis*. Mice lacking IL-17 failed to clear the bacteria from the nasal mucosae following infection with *B. pertussis* and these mice had defective production of CXCL1 and recruitment of Siglec-F^+^ neutrophils. Natural immunity induced by the previous infection was highly effective at preventing nasal infection with *B. pertussis*, however, rapid recruitment of Siglec-F^+^ neutrophils and prevention of re-infection was abrogated in the absence of functional IL-17.

Studies on T cell responses to *B. pertussis* have revealed that natural infection and immunization with wP vaccines induce Th1/Th17 responses, while aP vaccines preferentially induce Th2 cells in humans and animal models.^14,20–22,24–26^ The highly effective protection against re-infection of the lungs induced by natural infection has been linked to induction of Th1 cells, with IFN-γ playing a prominent role in protection in the lung by recruitment and activation of macrophages and opsonizing antibodies.^27,28^ The results from the present study reveal a distinct mechanism of protection in the nasal mucosa and provide evidence of a critical role for IL-17 secreted by Th17 cells. CD4 T_RM_ cells accumulate in the nasal tissue during infection with *B. pertussis* and these are predominantly IL-17 producing, with a small number secreting IFN-γ or both cytokines.

Previous studies had provided convincing evidence of a role for Th1 cells and IFN-γ in protection against *B. pertussis* infection in the lungs and while we do not rule out some role for IFN-γ-secreting T cells in protection against nasal infection, the present study demonstrated that IL-17 deficiency abrogated protection against primary infection or re-infection of the nasal mucosae. Low numbers of IFN-γ-producing T_RM_ cells accumulated in the nasal tissue during primary infection of WT mice with *B. pertussis*, and although these cells were significantly expanded in *Il17A*^−/−^ mice, these mice did not clear the infection in the nose for up to 60 days. Taken together with our previous reports,^28,34^ our findings demonstrate that while Th1 cells are key protective cells against lung infection, Th17 cells are more critical for protection against nasal infection. *B. pertussis*-specific IL-17-secreting T_RM_ cells persisted in nasal tissue for several months after bacterial clearance and expanded locally in the nasal tissue soon after secondary infection. Depletion studies with anti-CD4 antibody suggested that local T_RM_ cells mediate rapid clearance of the bacteria from the nasal tract. While our study does not rule out a contribution of other cell types, especially γδ T cells, our findings suggest that CD4 T cells are the primary source of IL-17, especially during re-infection of convalescent mice.

It has been reported that nasally delivered vaccines induce IL-17-secreting T_RM_ cells,^14,29,45^ however, this is the first study to demonstrate a central role for IL-17-secreting T_RM_ cells in protection against *B. pertussis* infection of the nasal mucosae. We have recently reported that, in an autoimmune disease model, IL-17 producing CD4 T cells recruit neutrophils through IL-17-mediated activation of chemokine production by epithelial cells.^47^ Therefore, during *B. pertussis* infection IL-17, secreted mainly by CD4 T_RM_ cells in the respiratory tract, may act on local epithelial cells to produce chemokines that recruit neutrophils. Indeed, we found significant production of CXCL1, a chemokine that promotes recruitment and activation of neutrophils, early in *B. pertussis* infection and this was reduced in *Il17A*^*−/−*^ mice. Furthermore, intranasal administration of IL-17 induced local CXCL1 production.

IL-17 has been linked with IgA-mediated protection in the nasal cavity of mice induced by attenuated vaccine *B. pertussis* strain BPZE1.^45^ Furthermore, circulating IgG induced by maternal immunization can prevent pertussis deaths in infants.^52^ However, we found significantly stronger *B. pertussis*-specific IgA and IgG2c antibody responses in lungs and nasal washes of *Il17A*^−/−^ mice compared with WT mice during primary or re-infection with *B. pertussis*. The higher IgA responses in *Il17A*^*−/−*^ mice is surprising, as it has been suggested that IL-17 has a positive effect on IgA production.^53^ The IgG2c response was also stronger in *Il17A*^−/−^ compared with WT mice and this is consistent with stronger Th1 responses in these mice. These data suggest that the IL-17-mediated protection against nasal infection with *B. pertussis* may not be mediated through antibody production. These findings are consistent with studies on other bacterial pathogens that have suggested that IL-17 mediates protective immunity against infection of the nasal mucosae or lungs independent of antibody production. It has been reported that Th17 cells play a role in immunity against nasal infection with *Streptococcus pneumonia;* protective immunity induced with a whole-cell vaccine was lost in *Il17AR*^−/−^ mice, but not in *Ifng*^*−/−*^ or *Il4*^*−/−*^ mice, and was antibody-independent.^30^ Interestingly, acquired immunity did not prevent initial infection with *S. pneumoniae*, but rather accelerated clearance upon re-challenge and this was associated with infiltration of neutrophils into the nasopharyngeal mucosa. Studies with *Staphylococcus aureus* have demonstrated clearance of the nasal infection is dependent on Th17 cells and IL-17A-mediated neutrophil recruitment.^32^ Control of *S. aureus* in the nose has also been linked to IL-17A and IL-17F dependent production of AMPs.^31^ Finally, intranasal vaccination with an attenuated strain of *Pseudomonas aeruginosa* induced antibody-independent, cross-serotype protection in the lung that was IL-17 dependent and linked to neutrophil recruitment.^33^ The findings of our study are consistent with these reports but demonstrate a key role for nasal tissue-resident neutrophils that express Siglec-F in nasal clearance of *B. pertussis* and acquired immunity against re-infection. We found Siglec-F^+^ neutrophils accumulated in the nasal tissue during primary infection and rapidly expanded following re-infection with *B. pertussis*. Infiltration of Siglec-F^+^ neutrophils mirrored the expansion of IL-17-producing *B. pertussis*-specific T_RM_ cells in nasal tissue and their recruitment was abrogated in *Il17A*^−/−^ mice or by neutralization of IL-17. Furthermore, Siglec-F^+^ neutrophils were significantly reduced in the nasal tissue of *Il17A*^*−/−*^ during infection with *B. pertussis* and this is associated with significantly higher bacterial burden in the nose. The absence of IL-17A had a less obvious effect on neutrophil recruitment or the course of infection in the lungs. Interestingly, Siglec-F^+^ neutrophils appear to have a propensity for recruitment to the nasal tissue at homoeostasis and during *B. pertussis* infection.

Neutrophil recruitment to the respiratory tract has been documented in humans, baboon and mice infected with *B. pertussis*.^54–58^ However, previous studies have suggested that neutrophils may not have a role in control of a primary infection with *B. pertussis*, but may have a role in antibody-mediated clearance of *B. pertussis* or *B. bronchiseptica* in immune mice.^41,59^ However, these studies focused on infection in the lungs and depleted neutrophils using the anti-GR1 antibody RB6-8C5, which recognizes Ly6G and Ly6C and the latter is also expressed on monocytes, macrophages, T cells and eosinophils.^42^ Depletion of neutrophils using the Ly6G-specific antibody 1A8 is also problematic as it is reliant on opsonization and phagocytosis by macrophages, a mechanism that is slower than the release of neutrophils from the bone marrow, especially in an acute infection.^43,60^ We adopted a novel depletion protocol, which includes a secondary antibody that binds 1A8, and switches the mechanism towards faster complement-mediated depletion.^43^ We found that neutrophil depletion with anti-Ly6G in combination with the secondary antibody had little effect on the bacterial load in the lung, but significantly impaired bacterial clearance from the nasal mucosae during primary infection or re-infection of convalescent mice with *B. pertussis*. Treatment of mice with the anti-Ly6G antibody did not have a consistent effect on conventional Siglec-F^-^ neutrophils, but significantly depleted Siglec-F^+^ neutrophils, especially in the nasal tissue. We do not have an explanation for why treatment of mice with the anti-Ly6G antibody has a more selective depleting effect on Siglec-F^+^ compared with Siglec-F^-^ neutrophils. Expression of CXCR2, the receptor for CXCL1 and induced by IL-17, CD49d, a component of VLA4 integrin involved in leukocyte migration and CD11c, integrin alpha-X involved in neutrophil adherence, are all higher on Siglec-F^+^ compared with Siglec-F^-^ neutrophils. Ly6G colocalizes with β2-integrins and their expression can be impaired by anti-Ly6G.^61^ Therefore, it is possible that expansion of Siglec-F^+^ neutrophils in the nasal tissue may be facilitated by higher expression of ligands that promote recruitment and adherence of Siglec-F^+^ neutrophils in that tissue. Overall, our data show that clearance of a primary or secondary infection with *B. pertussis* is reduced in anti-Ly6G treated mice and this has a significant effect on recruitment of Siglec-F^+^ neutrophils to the nasal cavity, providing evidence of the importance of Siglec-F^+^ neutrophils in clearance of *B. pertussis* infection of the nasal cavity.

Siglec-F^+^ neutrophils have been recently described in the nasal mucosa in an allergic rhinitis model and have an activated phenotype and enhanced effector function compared with conventional Siglec-F^-^ neutrophils.^35^ Furthermore, Ly6G^+^Siglec-F^+^ are present in the olfactory neuroepithelium of mice under steady-state conditions and increase during inflammation or tissue injury.^62^ We found very low numbers of Siglec-F^+^ neutrophils in the lung and other tissues, including the spleen, bone marrow, and NALT, but a high frequency in the nasal tissue in naive mice and this was significantly enhanced during nasal infection with *B. pertussis*. Although Siglec-F^-^ neutrophils also infiltrated the lungs and nasal tissue, only the infiltration of Siglec-F^+^ neutrophils was significantly reduced in *Il17A*^−/−^ mice following primary infection or re-infection with *B. pertussis*. We found that Siglec-F^+^ neutrophils had higher expression of CXCR2, CD11c and CD49d than Siglec-F^-^ neutrophils. CXCR2 is the receptor for CXCL1, which is produced in the respiratory tract during infection with *B. pertussis* in response to IL-17. CD11c is expressed on neutrophils during inflammation and sepsis, whereas CD49d, the integrin α subunit of VLA-4 is involved in migration of leukocytes. A pro-inflammatory role for Siglec-F^+^ neutrophils in respiratory tissue is consistent with data from an allergic rhinitis model^35^ and in a lung tumor model.^36^ We also found significantly lower CD44 expression on Siglec-F^+^ neutrophils. It has been shown in an *E.coli* pneumonia model^63^ and a *Klebsiella pneumoniae* model^64^ that accumulation of neutrophils in the lungs is reduced in *Cd44*^−/−^ mice. Neutrophils from *Cd44*^−/−^ mice also migrated further into a matrix expressing the CD44 ligand and extracellular matrix component hyaluronic acid.^63^ Since we found that almost all Siglec-F^+^ neutrophils in nasal tissue or lung tissue were tissue-resident (CD45 i.v. negative), it is possible that low expression of CD44 facilities migration of neutrophils into the respiratory tissue.

Siglec-F^+^ neutrophils showed a significantly higher capacity for NETosis than Siglec-F^−^ neutrophils and this was enhanced by stimulation with heat-killed *B. pertussis*, suggesting that Siglec-F^+^ neutrophils may have superior anti-bacterial function compared with Siglec-F^−^ neutrophils. Interestingly, CXCR2 signaling also induces NETosis in COPD^65,66^ and in tumors,^67^ providing support for our data showing that Siglec-F^+^ neutrophils are more predisposed to release NETs than Siglec-F^−^ neutrophils. Therefore, it is tempting to speculate that Siglec-F^+^ neutrophils play a role in protective immunity to *B. pertussis* in the nasal tissue through NETosis.

IL-17 has also been shown to induce antimicrobial peptide (AMP) production,^68,69^ and *B. pertussis* infection induces calprotectin (S100A8/A9) and the bacteriostaticum LCN2 in the lungs.^70^ We found enhanced *S100A8* and *Lcn2* gene expression in lungs and enhanced *Lcn2* expression in nasal tissue following re-challenge of convalescent mice with *B. pertussis*. Blocking IL-17 reduced the induction of *Lcn2* and *S100a8*. Since LCN2 is stored in neutrophil granules and released upon activation,^51,71^ it is possible that production of AMPs also contributes to the protective effect of neutrophils against nasal infection with *B. pertussis*.

Collectively, our data suggest that IL-17 plays an important role in the clearance of bacteria from the nasal cavity during infection with *B. pertussis* by promoting nasal tissue recruitment of neutrophils, especially Siglec-F^+^ neutrophils, which may help to eliminate the pathogen, possibly by NETosis and AMP production. The findings uncover a novel antibody-independent mechanism of acquired immunity in the nasal mucosae that involves neutrophil recruitment driven by IL-17-producing T_RM_ cells. Our study suggests that strategies to develop more effective pertussis vaccines that prevent nasal infection and asymptomatic transmission of *B. pertussis* need to consider the induction of IL-17-producing T_RM_ cells that recruit neutrophils to control infection of the nasal mucosae.

## Methods

### Animals

C57BL/6 and *Il17a*^*−/−*^ on a C57BL/6 background were bred in house from established colonies and housed in a specific pathogen-free facility in the Comparative Medicine Unit, Trinity College Dublin. *Il17a*^*−/−*^ and WT control mice were co-housed where indicated. Female mice were 6–12 weeks old at the initiation of experiments. All animal experiments were conducted in accordance to European Union regulations and under licence (AE19136/P042) from the Irish Health Products Regulatory Authority with approval from the Trinity College Dublin Bioresources Ethics Committee.

### *B. pertussis* respiratory challenge

*B. pertussis* bacteria were grown from a frozen stock for 3 days on Bordet-Gengou plates containing glycerol and horse blood (Cruinn). Bacteria were then collected in supplemented Stainer-Scholte medium and cultured overnight at 37 °C in a shaking incubator at 220 rpm. Bacteria were centrifuged and resuspended in 1% casein solution, and the OD was measured at 600 nm .^70^ Mice were infected by aerosol challenge (BP338 strain) administered using a nebulizer (PARI TurboBOY SX) from a culture at 1 × 10^9^ CFU/mL over 10 min as described previously.^72^ The course of infection (or re-infection) was followed by performing CFU counts on nasal washes and lung homogenates at intervals post challenge. Trans-pharyngeal nasal washes were collected as described .^73^ Briefly, 700 μl of sterile physiological saline was flushed through the exposed nasopharynx of euthanized mice, and collected when emerging from the nares. The left and post-caval lung lobes were aseptically removed and homogenised in 500 µl of sterile physiological saline with 1% casein. Undiluted and serially diluted homogenate (100 µl) and nasal wash (100 μl) was spotted onto Bordet-Gengou agar plates, and CFU counts were enumerated 5 days after incubation at 37 °C.

### In vivo treatment with antibodies

For IL-17A neutralization, mice were injected i.p. with anti-IL-17 Ab (17F3; BioXcell) at 200 μg/mouse 1 d prior to infection and every 3 days after challenge. A double antibody-based depletion strategy adapted from Boivin et al. was employed for neutrophil depletion .^43^ Briefly, mice were injected i.p. with anti-Ly6G Ab (1A8; BioXcell; 50 μg/mouse) 1 d prior to and every day after challenge. The dose of anti-Ly6G Ab was increased to 100 μg/mouse from day 9 post-infection onwards in convalescent mice and from day 4 in naive mice. A secondary Ab, anti-rat Kappa immunoglobulin light chain (MAR 18.5, BioXcell) at 100 μg/mouse was injected i.p every second day after anti-Ly6G treatment. The dose of secondary antibody was increased to 150 μg/mouse in-line with the anti-Ly6G-dose increase. For depletion of neutrophils during an active *B. pertussis* infection, mice were injected i.p. with anti-Ly6G Ab (100 μg/mouse) every day from day 7 post-challenge. Anti-rat Kappa immunoglobulin (100 μg/mouse) was injected i.p every second day after anti-Ly6G treatment. Corresponding isotype controls were used (Mouse IgG1, κ; Rat IgG2a, κ; BioXcell). For neutrophil depletion, mice treated with isotype control antibodies also received anti-rat Kappa immunoglobulin.

For depletion of CD4 T cells during a primary *B. pertussis* challenge, mice were treated with anti-CD4 Ab (GK1.5; BioXcell), 200 μg/mouse i.p. and 50 μg/mouse i.n. simultaneously, every 4 days starting at 7 days after challenge. For depletion of CD4 T cells during the re-challenge of convalescent mice, convalescent mice were treated with anti-CD4, 200 μg/mouse i.p. and 50 μg/mouse i.n., 5 and 1 day before and every 4 days after secondary *B. pertussis* challenge. A corresponding isotype antibody (Rat IgG2b, κ; BioXcell) was used as a control.

### Intranasal administration of IL-17

Mice were anesthetised by sub-cutaneous injection of ketamine/xylazine and received 500 ng/mouse of recombinant murine IL-17A (Immunotools) in a volume of 40 µl PBS (20 µl per nares) by the i.n. route. Control mice received 40 µl PBS.

### Detection of respiratory tissue-resident immune cells

Mice were injected intravenously (i.v.) with 1.5 µg of PE-conjugated anti-CD45 antibody (Clone 30-F11; eBioscience) in 200 μl PBS 10 min prior to euthanasia. Circulating lymphocytes are exposed to the antibody and are labeled CD45 i.v.^+^, while tissue-resident lymphocytes remain unlabeled. thus are CD45 i.v.^−^.

### Isolation and flow cytometry analysis of cells from nasal tissue and lungs

The nasal tissue, including the nasal cavity and nasal turbinates, was obtained as described.^74^ The upper palate, which contains the nasal-associated lymphoid tissue (NALT), was separated from the upper jaw. Three lung lobes (superior, middle and inferior) were isolated and chopped. Both nasal tissue and lung were digested with collagenase-D (1 mg/mL; Roche) and DNase I (10 mg/mL; Sigma-Aldrich) for 1 h at 37 °C with agitation. Nasal tissue, upper palate, lung and spleen were passed through a 70-µm cell strainer to a obtain single-cell suspension. A single cell suspension was obtained from bone marrow by flushing femurs and tibiae with RPMI using a 25-gauge needle. Ammonium-chloride-potassium (ACK) buffer was used to lyse red blood cells. Single cell suspension was washed with PBS, incubated with LIVE/DEAD Aqua (1:600) (Invitrogen), followed by incubating with Fc block (αCD16/CD32 FcγRIII) (1:200) (BD Biosciences) to block IgG Fc receptors. Surface markers were then stained with flurochrome-conjugated anti-mouse antibodies and fixed with 2% paraformaldehyde (PFA, ThermoFisher Scientific). For detection of *B. pertussis*-specific intracellular cytokines, cells were stimulated for 18–20 h at 37°C in 5% CO_2_ with sonicated *B. pertussis* (sBP; 5 μg/mL), anti-CD28 and anti-CD49d (1 μg/mL each; BD Biosciences), with brefeldin A (5 µg/mL; Sigma-Aldrich) added for the final 4 h of incubation. Alternatively, intracellular cytokine production was also detected following stimulation of cells with PMA (50 ng/ml) and ionomycin (500 ng/ml) in the presence of brefeldin A (5 μg/ml) for 4 h at 37°C. For detection of intracellular cytokines and transcription factors, cells were fixed, permeabilized and stained using eBioscience^TM^ Foxp3/Transcription Factor Staining Buffer Set (ThermoFisher Scientific) according to the manufacturer’s instructions. The antibodies for surface and intracellular cytokine staining are listed in Supplementary Table S1. Fluorescence minus one (FMO) samples were used as controls. Flow cytometric analysis was performed on an LSRFortessa or a Cytek Aurora, and data were acquired using Diva software (BD Biosciences) or SpectroFlo^®^ software (Cytek Biosciences), respectively. Data were analyzed using FlowJo software (Tree Star).

### Enzyme-linked immunosorbent assay (ELISA)

Levels of CXCL1 (R&D System) from nasal washes and lung homogenate supernatants were measured by ELISA, according to the manufacturer’s instructions and read using a VersaMax microplate reader (Molecular Devices).

### Detection of NET activity by flow cytometry

We employed an established technique using a combination of the cell-impermeable nucleic acid dye, SYTOX Green, and 4’,6-diamidino-2-phenylindole (DAPI), which is membrane-semi-permeable, for two-colour visualization of NETs on flow cytometry.^75,76^ Single cell suspensions were obtained from nasal tissue and added to U-bottom 96-well polypropylene plates. Cells were incubated for 30 min at 37 °C in 5% CO_2_ prior to stimulating with heat-killed *B. pertussis* (1 × 10^7^ CFU/mL), PMA (50 ng/mL; Sigma-Aldrich), or medium for 4 h. Cells were fixed by adding PFA at a final concertation of 1.3% and incubated for 15 min at RT. PFA concentrations below 4% are thought to fix, but not permeate the plasma membrane, thus reducing artificial “NET formation” .^76^ Samples were then centrifuged at 100 × *g* for 10 min at RT and supernatants were carefully aspirated. Samples were stained with the following: DAPI (0.1 µg/mL; Sigma-Aldrich), SYTOX Green (0.3 µM; Thermo-Fisher Scientific), CD11b-APCeFlour780 (MEL-14; eBioscience), Ly6G-BV650 (1A8; BioLegend), and Siglec-F-PECF594 (E50-2440; BD Biosciences).

### Detection of B. pertussis-specific antibodies

*B. pertussis*-specific antibodies in individual mouse nasal washes and lung homogenate supernatants were quantified by ELISA using plate-bound sBP (1 µg/mL), biotin-conjugated anti-mouse IgA (1:1500; Southern Biotech) or IgG2c (1:1500; Southern Biotech) and peroxidase-conjugated streptavidin. The reaction was developed using TMB and 1 M H_2_SO_4_ and the plates were read using a VersaMax microplate reader (Molecular Devices) at 450 nm.

### RT-qPCR

Nasal tissue or lung tissue was collected into RNAlater solution. RNA was subsequently isolated using TriZol Reagent (Invitrogen) and reverse transcribed in cDNA using High Capacity cDNA Reverse Transcription Kit (Applied Biosystems). qRT-PCR was performed using commercially available probes (Thermo Fisher Scientific) *Cxcl1* (Mm04207460_m1), *Cxcl2* (Mm00436450_m1), *Csf3* (Mm00438334_m1), *Il1b* (Mm00434228_m1), *Lcn2* (MM01324480_m1), and *S100a8* (MM00496696_g1). qRT-PCR was performed on a PRISM7500 Sequence Detection System (ABI). The amount of each gene was determined by normalization to 18S rRNA (Mm04277571, ABI) internal control.

### Statistical analysis

Statistical analysis was performed using Prism 8 (GraphPad Software). Data were analyzed using one- or two-way ANOVA, followed by the multiple comparison tests, or a two-tailed unpaired *t* test, as appropriate. Data are expressed as mean with SEM. *P*-values less than 0.05 were considered significant.

## Supplementary information

Supplementary Materials

Supplementary Table S1
