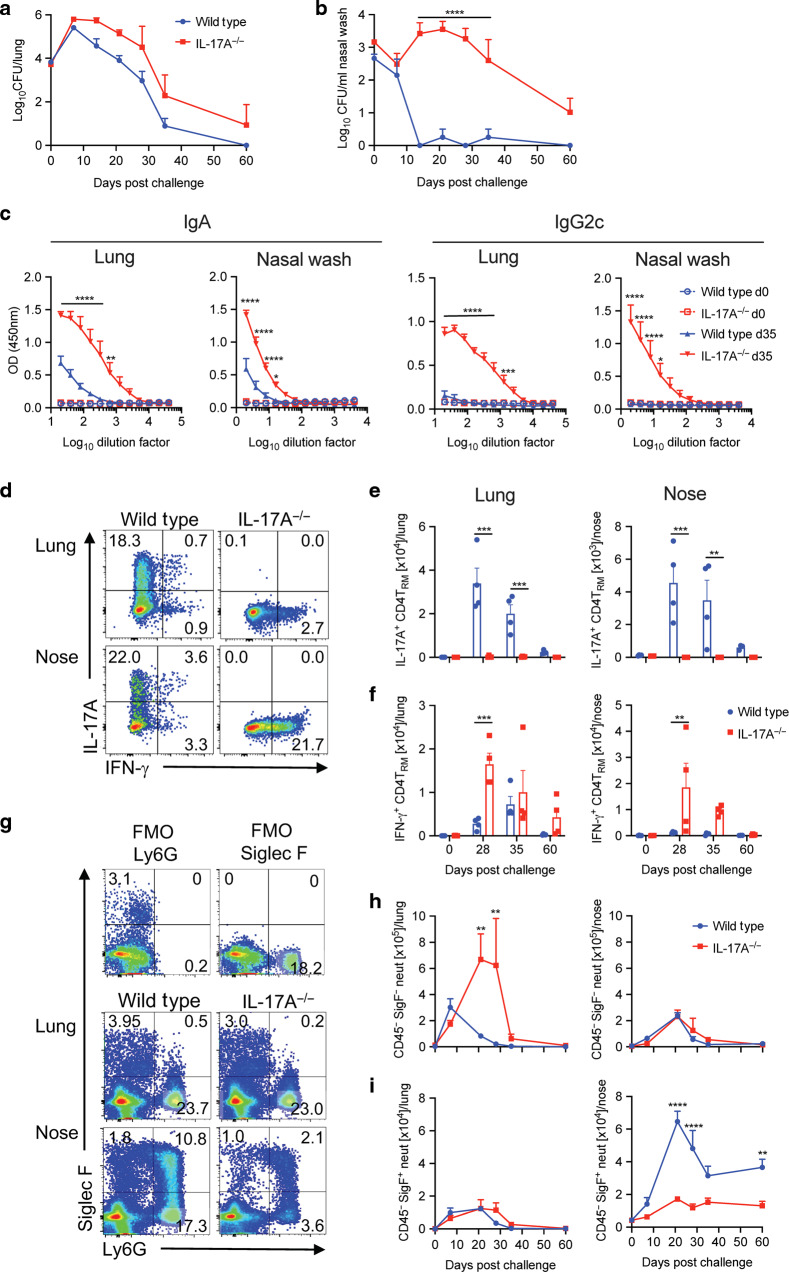# Correction: IL-17 mediates protective immunity against nasal infection with *Bordetella pertussis* by mobilizing neutrophils, especially Siglec-F^+^ neutrophils

**DOI:** 10.1038/s41385-021-00417-3

**Published:** 2021-06-02

**Authors:** Lisa Borkner, Lucy M. Curham, Mieszko M. Wilk, Barry Moran, Kingston H. G. Mills

**Affiliations:** Immune Regulation Research Group, School of Biochemistry and Immunology, Trinity Biomedical Sciences Institute, Trinity College Dublin, Dublin, Ireland

Correction to: *Mucosal Immunology* 10.1038/s41385-021-00407-5

The original version of this article unfortunately contained a mistake in Fig. 3h. Due to a typesetting error the lines on the graph have been moved below the X axis. We apologize for this error. The correct Fig. 3h can be found below. The original article has been corrected.

**Figure:**