# Supplementary material for: IL-17 mediates protective immunity against nasal infection with Bordetella pertussis by mobilizing neutrophils, especially Siglec-F+ neutrophils
Source: Mucosal Immunol. 2021 May 11;14(5):1183–202. doi: 10.1038/s41385-021-00407-5 (PMC8379078; doi:10.1038/s41385-021-00407-5)
Supplement: Supplementary file 1 — Supplementary Materials [file 41385_2021_407_MOESM1_ESM.pdf]

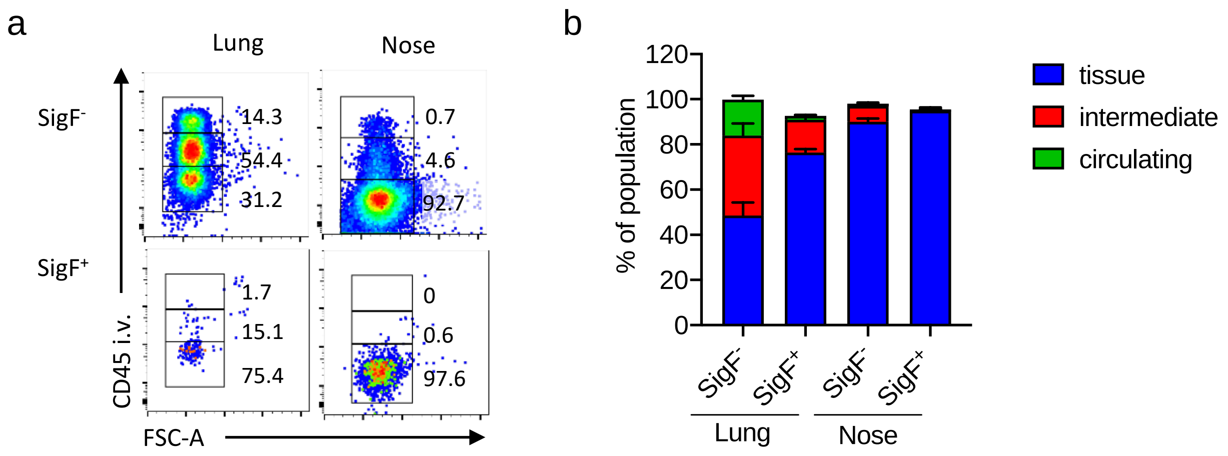

Supplementary Fig. 1 Siglec-F^{+} neutrophils are predominately tissue-resident. C57BL/6 mice were aerosol infected with *B. pertussis*. 7 days post challenge, mice were injected i.v. with fluorochrome-labelled CD45 antibody and euthanized 10 min later. Cell suspensions were prepared from lung and nasal tissue and immune cells were analysed by flow cytometry. **a** Representative dot plots showing the distribution of the CD45 in vivo staining for Siglec-F^{-} or Siglec-F^{+} neutrophils. **b** Percentage of tissue (CD45 i.v.$^{-}$), intermediate (CD45 i.v.^{inter}) and circulating (CD45 i.v.$^{+}$) cells within the Siglec-F^{-} and Siglec-F^{+} neutrophil population in lung and nose. n=5, mean ± SEM

a

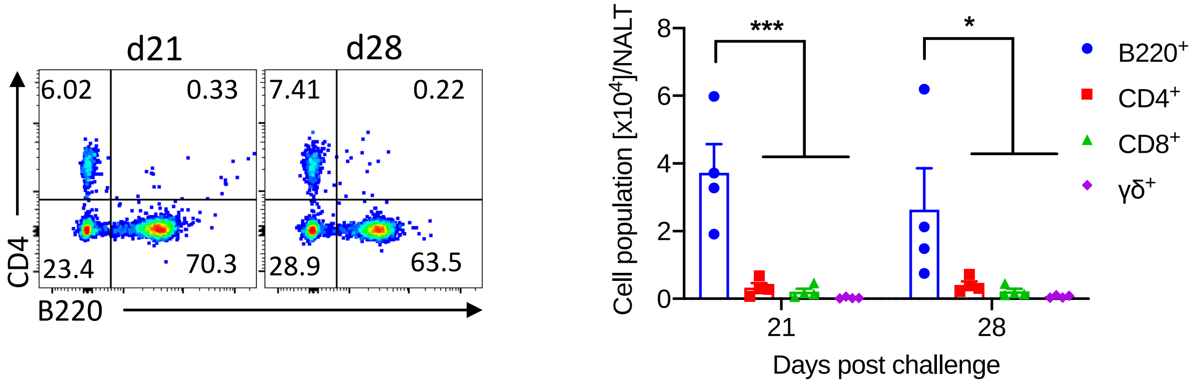

b

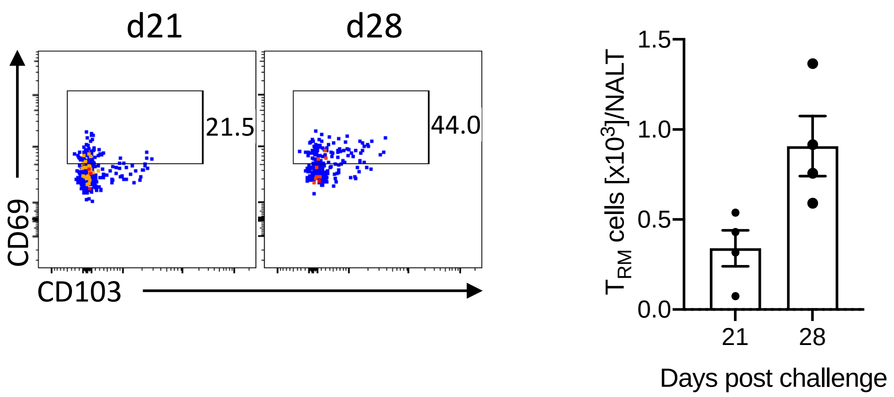

Supplementary Fig. 2 B cells are the dominant cell population in NALT during *B. pertussis* infection. C57BL/6 mice were aerosol infected with *B. pertussis*. On 21 or 28 d post infection mice were injected i.v. with fluorochrome-labelled CD45 antibody and euthanized 10 min later. Cell suspensions were prepared from NALT tissue and were analysed by flow cytometry. **a** Representative dot plots showing CD4 and B220 expression on NALT cells. Absolute counts of CD4, CD8, γδ T cells and B cells in NALT. **b** Representative dot plots of CD69 and CD103 expression on CD45 i.v.$^{-}$ CD44^{+} CD4 T cells in NALT. Absolute counts of T_{RM} cells in NALT. Statistical Analysis: Two-way ANOVA followed by Tukey's post-test, *$p < 0.05$, ***$p < 0.001$, n=4/group, mean ± SEM

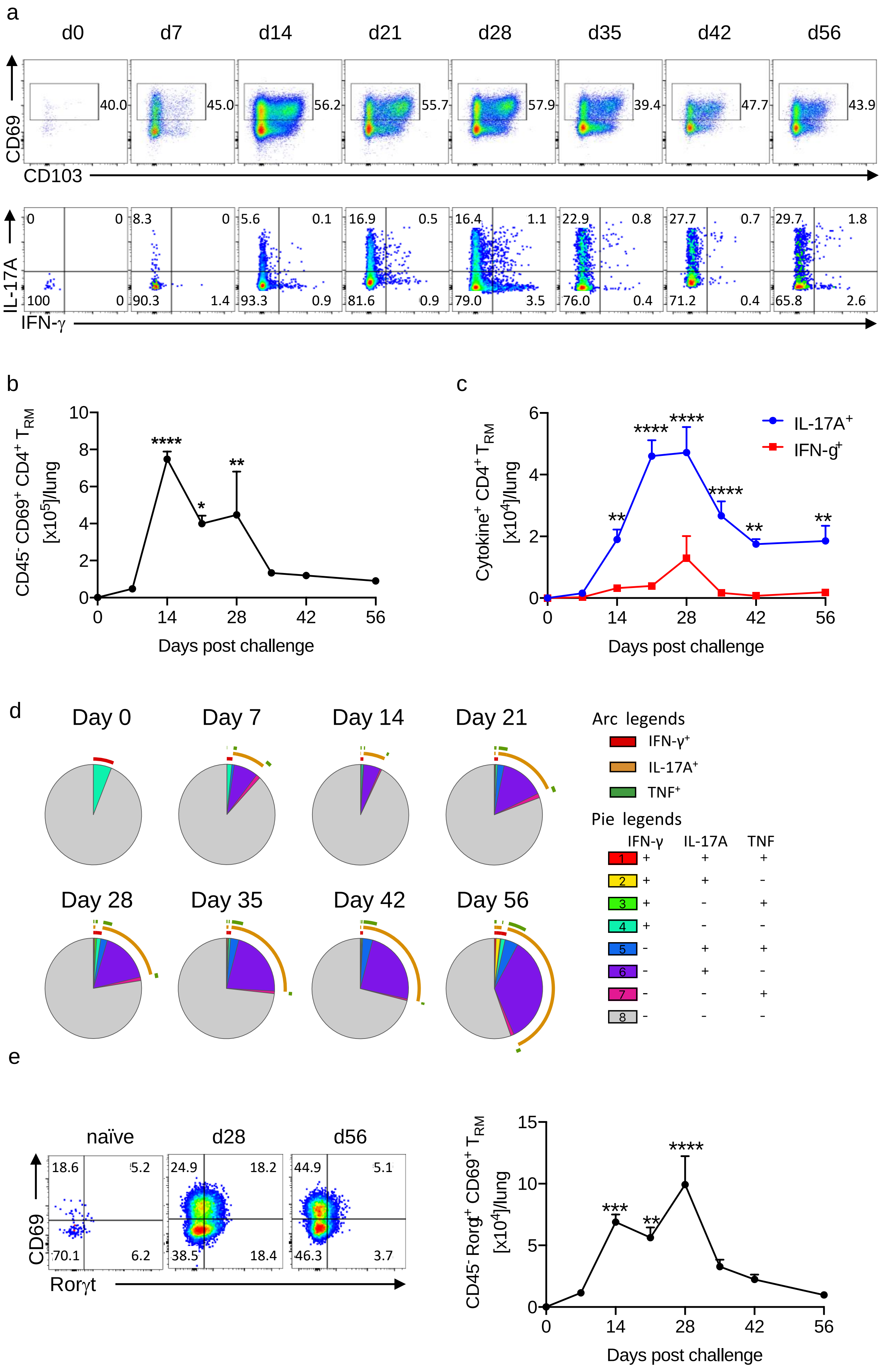

a
d0
d7
d14
d21
d28
d35
d42
d56
CD69
CD103
40.0
45.0
56.2
55.7
57.9
39.4
47.7
43.9
IL-17A
IFN-γ
b
CD45- CD69+ CD4+ TRM [x10^5]/lung
Days post challenge
c
Cytokine+ CD4+ TRM [x10^4]/lung
IL-17A+
IFN-g+
Days post challenge
d
Day 0
Day 7
Day 14
Day 21
Day 28
Day 35
Day 42
Day 56
Arc legends
IFN-γ+
IL-17A+
TNF+
Pie legends
IFN-γ
IL-17A
TNF
e
naïve
d28
d56
CD69
Rorγt
CD45- Rorgt+ CD69+ TRM [x10^4]/lung
Days post challenge

Supplementary Fig. 3 Accumulation of CD4 T_{RM} cells in the lungs during *B. pertussis* infection. C57BL/6 mice were aerosol infected with *B. pertussis*. At different time points, mice were injected i.v. with fluorochrome-labelled CD45 antibody and euthanized 10 min later. Cell suspensions were prepared from lung tissue and cells were stimulated with sBP and anti-CD49d and antii-CD28 and analysed by flow cytometry. **a** CD69 and CD103 expression on tissue resident $CD44^+$ $CD4^+$ T cells and IL-17A and IFN-γ-production in the $CD69^+$ sub-population. **b** Numbers of CD4 T_{RM} (CD45i.v.$^-$ $CD4^+$ $CD44^+$ $CD69^+$) in lung tissue during *B. pertussis* infection. **c** Numbers of IL-17A- and IFN-γ- producing CD4 T_{RM} cells. **d** SPICE analysis of cytokine production in sBP stimulated CD4 T_{RM}. Cells. **e** Analysis of RORγT expression in CD4 T_{RM} cells in naïve mice and 28 and 56 days post infection. Absolute numbers of tissue-resident $ROR\gamma T^+$ $CD69^+$ CD4 T cells. Statistical analysis: **b** One-way ANOVA followed by Dunnett's post-test, significances are indicated in comparison to naïve mice; **c** Two-way ANOVA followed by Sidak's post-test, significances are indicated in comparison to naïve mice; $*p < 0.05$, $**p < 0.01$, $***p < 0.001$, n=4/group, mean ± SEM

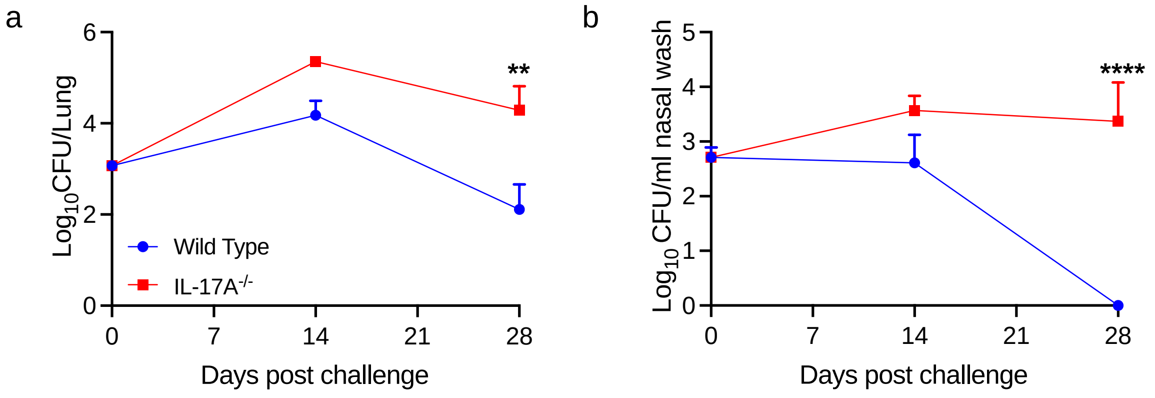

Supplementary Fig. 4 Enhanced *B. pertussis* infection in IL-17A$^{-/-}$ mice. WT and *Il17A*$^{-/-}$ mice were co-housed prior to and during *B. pertussis* infection. On days 14 and 28 post aerosol infection, mice were sacrificed. CFU counts in lungs **(a)** and nasal wash **(b).** Statistical analysis: Two-way ANOVA followed by Sidak's post-test, **p* < 0.05, *****p* < 0.0001, n=4/group, mean ± SEM

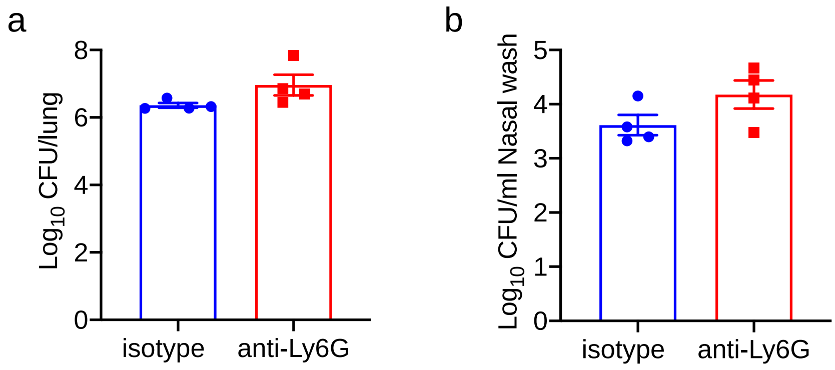

Supplementary Fig. 5 Depletion of neutrophils does not affect bacterial load in lungs or nasal mucosae of *IL17A*$^{-/-}$ mice. *IL17A*$^{-/-}$ mice were treated with anti-Ly6G and with secondary antibody MAR 18.5 from at d -1 of infection, and aerosol infected with *B. pertussis*. 10 days post infection, mice were euthanised. CFU counts in lung **(a)** and nasal washes **(b).**

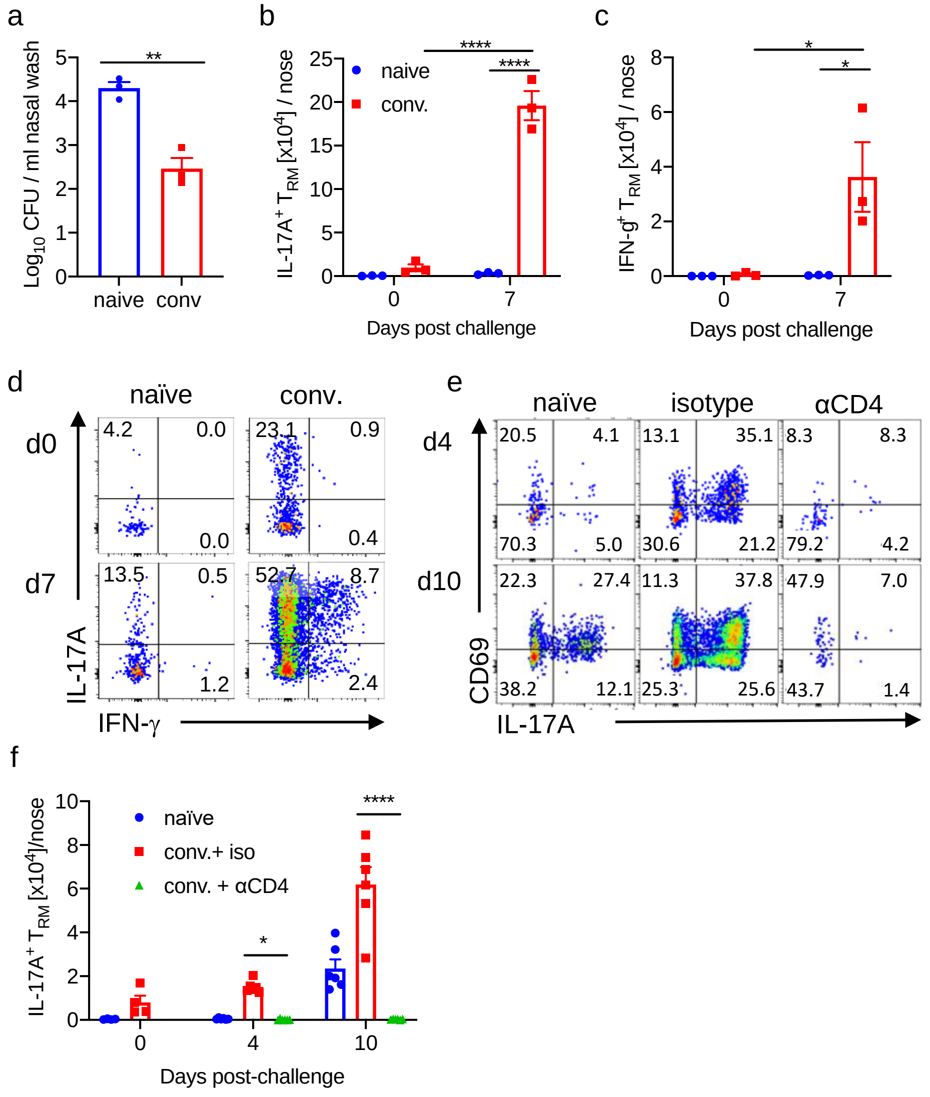

Supplementary Fig. 6 CD4 T_{RM} cells expand in nasal tissue after re-infection with *B. pertussis*. C57BL/6 mice were aerosol infected with *B. pertussis* and allowed to clear the infection. One year later, mice were re-challenged with *B.* pertussis together with naïve controls. Mice were euthanized at 7 d post challenge, cell suspensions were prepared from lung and nasal tissue and immune cells were analysed by flow cytometry. **a** CFU counts in lung and nasal washes of naïve and convalescent mice on day 7 post *B. pertussis* challenge. **b** IL-17- and **c** IFN-γ-producing CD4 T_{RM} cells in nasal tissue before and 7 days after re-infection. **d** Representative dot plots showing IFN-γ and IL-17A expression in CD4 T_{RM} cells. C57BL/6 mice were aerosol infected with *B. pertussis* and allowed to clear the infection. 71 days later, mice were treated with anti-CD4 antibody or isotype control starting from d -1 of infection. 7 and 10 days post aerosol infection, mice were injected i.v. with fluorochrome-labelled CD45 antibody and euthanized 10 min later. Cell suspensions were prepared from lung and nasal tissue and immune cells were analysed by flow cytometry. **e** Representative dot plots for CD69 and IL-17A expression in tissue-resident (CD45 i.v. negative) $CD44^+$ CD4 T cells. **f** Absolute counts of IL-17 producing CD4 T_{RM} cells in nasal tissue. Statistical analysis: Two-way ANOVA followed by Sidak's post-test, *: p value < 0.05, ***: p value < 0.001, ****: p value <0.0001. **a-c** n=3/group, **f** pooled data from two independent experiments n=6-10/ group mean ± SEM
